# Changes in circulating NK and innate-like T cells in type 1 and type 2 diabetes

**DOI:** 10.3389/fimmu.2025.1667888

**Published:** 2025-12-04

**Authors:** Marina Loguinova, Nikita Sergeev, Margarita Samsonova, Alyona Sorokina, Dmitry Grebennikov, Ivan Golodnikov, Anna Goncharenko, Gennady Bocharov, Dmitry Laptev, Rita Khusainova, Ildar Minniakhmetov, Marina Shestakova, Ivan Dedov, Natalia Mokrysheva

**Affiliations:** 1 Endocrinology Research Centre, Moscow, Russia; 2 Marchuk Institute of Numerical Mathematics, Russian Academy of Sciences (INM RAS), Moscow, Russia; 3 Moscow Center of Fundamental and Applied Mathematics at INM RAS, Moscow, Russia; 4 Sechenov First Moscow State Medical University of the Ministry of Health of the Russian Federation (Sechenov University), Moscow, Russia

**Keywords:** type 1 and type 2 diabetes mellitus, autoimmune inflammation, NK cells, NK8+, CD38, CD161, NKG2A, innate-like T cells

## Abstract

**Background:**

Inflammatory responses that accompany the progression of type 1 (T1D) and type 2 diabetes mellitus (T2D) are fundamentally distinct in their underlying nature. T1D is predominantly driven by autoimmune-mediated inflammation, whereas T2D is characterized by a chronic, low-grade metabolic inflammation. A growing body of evidence has highlighted the involvement of natural killer (NK) cells in pathophysiology of both forms of diabetes; nevertheless, the precise mechanisms and the roles played by specific NK cell subsets remain incompletely understood.

**Methods:**

Multicolor flow cytometry was used to identify several NK and innate-like T cell subpopulations in peripheral blood of patients with adult-onset T1D (n=23) and T2D (n=14) in comparison to healthy volunteers (n=24). Subset identification was based on expression of functional antigens (CD16 and CD56), co-receptors (CD8 and CD38), inhibitory receptors (NKG2A and CD161), and transcription factor EOMES. Quantitative analysis using Spearman’s rank correlation coefficients was performed to identify possible association between immune and clinical parameters and to rank the clinical parameters with respect to the number of connections with immune cell populations.

**Results:**

T1D was accompanied by a reduction in overall NK cells and their dominant cytolytic CD56^dim^CD16^bright^ subpopulation. Also a 50% increase in frequency of CD8+ NK cells was observed together with a growth of CD8+CD38+, CD8+CD161+, CD8+NKG2A+, and CD8+EOMES+ NK subsets. T2D was associated with a more than twofold decrease in the frequency of MAIT cell subset within CD8+ T cell population. Correlation analysis of the obtained data set revealed quantitative relationships between immune status parameters and clinical indicators, highlighting the involvement of NK cell subsets and innate-like T cells in shaping the phenotype of the pathological process and their complex role in regulating the immunopathogenesis of T1D and T2D.

**Conclusion:**

In this pilot study, we provide evidence that different subpopulations of NK cells and innate-like T cells are involved in the immunopathogenesis of T1D and T2D, through a decline in the control of immune reactivity in T1D, while sustaining chronic metainflammatory responses in T2D. A critical elevation of CD8+ NK cells was associated with T1D, while loss of circulating MAIT cells was a hallmark of T2D.

## Introduction

Diabetes mellitus (DM) constitutes a metabolic disorder typified by disturbances in glucose and lipid homeostasis. Type 1 DM (T1D) represents a classic autoimmune disease, distinguished by immune reactivity directed against self-antigens of pancreatic β-cells, ultimately resulting in their progressive destruction (1, 2). In contrast, type 2 diabetes (T2D) is a multifactorial and heterogeneous metabolic condition, the pathogenesis of which is frequently attributed to a complex interplay of obesity, insulin resistance (IR), and β-cell dysfunction (3), leading to a decline in insulin secretion and/or insulin sensitivity (4).

The aetiopathogenesis of T1D involves intricate crosstalk between pancreatic β-cells and both innate and adaptive immune cell populations (5, 6). Insights from experimental models have facilitated the identification of pivotal cellular and molecular mechanisms, confirming the central role of autoreactive T lymphocytes in the immunological assault on β-cells (6–8). Accordingly, the majority of T1D research has traditionally focused on diabetogenic T cell subsets and their modulation by regulatory T cells. Nevertheless, accumulating evidence underscores a critical role for innate immune effectors, including natural killer (NK) cells, in the orchestration and perpetuation of autoimmune responses (6, 9–11). NK cells have been found to infiltrate the pancreas at earlier stages than T lymphocytes and exhibit an exhausted, hypo-functional phenotype in the T1D context (12). The role of NK cells in T1D remains contentious. It is not yet fully resolved whether these cells function as drivers of β-cell destruction, or conversely, if they exert protective, immunoregulatory functions that mitigate autoimmune aggression (13, 14). An alternative hypothesis posits that their role may shift depending on disease stage and microenvironmental cues. In support of a pathogenic function, several murine studies have shown that NK cell depletion reduces insulitis and promotes remission of T1D (9, 15). Furthermore, NK cell frequency and activation status within pancreatic infiltrates have been shown to correlate with the extent of tissue destruction (7). Direct cytotoxic activity against β-cells that express ligands for NK cell activating receptors, NKp46 and NKG2D (e.g. RAE1, retinoic acid early transcript 1), has also been demonstrated (16). Beyond direct cytotoxicity, NK cells may exert indirect immunomodulatory effects that shape T1D progression. Through cytokine production, they can influence the activation and recruitment of diabetogenic CD4+ T cells (15), while potentially suppressing the activity of regulatory T cells (Tregs) (17). NK cells are capable of selectively lysing immature dendritic cells (DCs) (18); by sparing mature immunogenic DCs, they may promote CD8+ T cell priming and clonal expansion, thereby enhancing cytotoxic immune responses (19). The existence of the intricate cross-talk between NK cells, DCs and metabolic status of β-cells is demonstrated by Bode et al. in a rodent mouse model (20). Conversely, other studies have attributed to NK cells a protective function through suppression of cytotoxic CD8+ T cell proliferation (21, 22).

Human studies, although more limited in scope, report alterations in the frequency and function of peripheral NK cells in T1D. However, data remain inconsistent (13, 23). Some studies describe a reduced proportion of NK cells in newly diagnosed patients (24), whereas others report no significant change (17). Divergent results have also been reported concerning NK cell cytotoxicity: some investigators note diminished effector function (24), while others document heightened cytotoxic potential (24). A reduction in NKG2D expression appears to occur irrespective of disease duration (25). In long-standing T1D, NK cells exhibit diminished function, with reduced expression of activating receptors such as NKp30 and NKp46, and lower production of IFNγ and perforin, likely reflecting metabolic dysregulation, effects of insulin therapy, and progressive cellular exhaustion (24, 26). It is plausible that NK cells interact with the autoimmune milieu in a phase-dependent manner. During early disease stages, they may be recruited to the pancreas and act as cytotoxic effectors against β-cells. By releasing cytokines such as IFNγ within the pancreatic lymph nodes and islets, they could enhance activation of antigen-presenting cells (APCs) and trigger antigen-specific T cell responses (27). Thus, in the initial phase, NK cells might function as proinflammatory effectors that promote and sustain autoimmunity. At later stages, they may become functionally exhausted or suppressed by other immune cell types (12).

The pathophysiological basis of obesity-associated T2D is closely linked to insulin resistance (IR) and a persistent, low-grade inflammatory state originating in visceral adipose tissue (VAT), which progressively affects other insulin-sensitive organs including liver and skeletal muscle (28). Glucotoxicity and lipotoxicity contribute further to pancreatic inflammation by promoting the local release of proinflammatory cytokines such as IL-1β, IL-6 and IL-8, leading to β-cell dysfunction and impaired insulin secretion (29). Systemic inflammation is sustained by hyperglycaemia and driven by immune cell infiltration into adipose tissue, where neutrophils, macrophages and lymphocytes orchestrate the production of inflammatory mediators (30). Despite these insights, the precise cellular contributors to T2D pathogenesis, including NK cells and their various subpopulations (31, 32) remain only partially defined. CD56^bright^ NK cell proliferation and activation within adipose tissue have been shown to play a pivotal role in promoting IR and the transition to overt T2D (33–35). These cells, through IFNγ secretion, can recruit macrophages and induce their polarization towards a proinflammatory M1 phenotype, thereby enhancing the production of cytokines such as IL-6 and TNFα and reinforcing the inflammatory state in VAT (33, 36). Nevertheless, the functional contribution of specific NK cell subsets, including NKT cells, to the accumulation and activation of proinflammatory macrophages in adipose tissue remains insufficiently understood (37).

Peripheral blood NK cells constitute a minor subpopulation of lymphocytes (8–18%), belonging to the family of innate lymphoid cells (ILCs) endowed with cytotoxic capabilities. While classically known for their ability to eliminate stressed cells, such as virally infected or oncogenically transformed targets, NK cells are also capable of exerting cytotoxic effects on immune cells themselves, thereby regulating the overall immune response. Unlike T and B lymphocytes, NK cells do not undergo receptor gene rearrangement and hence lack the antigen-specific receptor diversity characteristic of adaptive immunity. Instead, NK cell heterogeneity is governed by the combinatorial expression of activating and inhibitory surface receptors that collectively define their functional phenotype. The effector behavior of NK cells, particularly in relation to immune targets, is finely tuned and driven not only by the cytokine environment but the balance between activating and inhibitory receptors. Among these, NKG2A serves as a critical inhibitory receptor that maintains immune tolerance. Another receptor, CD161 (NKR-P1A), expressed on most NK cells, suppresses their cytotoxic function following interaction with its ligand lectin-like transcript 1 (LLT1), which is expressed on activated APCs (38). This interaction serves to protect APCs from NK cell-mediated lysis, thereby permitting continued T cell activation. The biology of CD8 expression on NK cells remains incompletely understood. CD16+CD8+CXCR3+ NK cells have been found to be elevated in autoantibody-positive individuals who later progressed to overt T1D (39). In addition, increased frequencies of CD3-CD8^dim^CD56+ NK cells, which exhibit memory-like responses to GAD65 peptide epitopes, have been reported in patients at disease onset (40).

In the present pilot study, we employed multicolor flow cytometry to investigate circulating NK and innate-like T cells in small cohorts of patients with T1D and T2D in comparison to healthy donors. We aimed to measure the frequency of multiple NK and innate-like T cell subsets to evaluate functional potential of circulating NK and NKT cells to elucidate their role in the immunopathogenesis of both T1D and T2D. We determined NK subsets via CD16 and CD56 expression, by co-expression of functional modulators such as the inhibitory receptors NKG2A and CD161, co-receptors CD8 and CD38, and the transcription factor EOMES. Innate-like T cell subsets including invariant NKT (iNKT), and mucosa-associated invariant T cells (MAIT) were also identified. Changes in frequency of circulating NK and NKT cell subsets in T1D and T2D could reflect their critical role in the aetiopathogenesis of diabetes mellitus. The characterization of these changes may contribute in identifying potent biomarkers for developing novel, targeted therapeutic strategies aimed at mitigating diabetes mellitus and its associated complications.

## Materials and methods

The study was conducted at the Endocrine Research Centre from February 2023 to March 2025.

### Study subjects

A total of 61 participants were included and divided into three groups: healthy volunteers (n = 24), patients with T1D (n = 23) with disease duration up to one year, and patients with T2D (n = 14) with disease duration up to five years. Adult-onset T1D was defined as insulin-requiring diabetes at diagnosis or within ≤6 months plus evidence of autoimmune aetiology and/or β-cell failure. T2D was defined by clinician diagnosis consistent with guideline-based criteria, absence of insulin requirement during the first 6 months after diagnosis, and no evidence of islet autoimmunity. Inclusion criteria were as follows: age between 18 and 55 years, body mass index (BMI) no greater than 35 kg/m², and presence of one of the following conditions: T1D of less than one year in duration, or T2D of no more than five years. All patients with T2D except two of them had ≤1 year of disease. Exclusion criteria included the presence of another systemic autoimmune disease (based on medical records), any pancreatic disorder or prior pancreatic surgery (according to medical documentation), as well as the use of immunosuppressive therapy within the previous 12 months (as confirmed by medical history). Healthy volunteers were enrolled in the study based on predefined eligibility criteria analogous to those used for the diabetes patient groups. Participants included men and women aged 18 to 55 years with a BMI below 35 kg/m². Individuals with a confirmed diagnosis of any type of diabetes, including impaired glucose tolerance or impaired fasting glycaemia, were excluded. Participants were recruited using a consecutive sampling method, including all patients meeting the criteria who were receiving either inpatient or outpatient care during the study period. The study was observational, cross-sectional and conducted at a single center.

The local ethics committee of the Endocrine Research Centre approved the conduct of this study (extract from minutes No. 18 dated 12 October 2022). Written informed consent was obtained from all participants.

### Measurement of the clinical parameters of the study participants

The groups comprising patients with T1D, T2D and healthy volunteers differed in age, which was anticipated given the clinical characteristics of disease onset, as T1D typically manifests at a younger age compared to T2D. Patients with T2D were older than those with T1D and the healthy control group (**Table 1**). HbA1c levels and fasting plasma glucose did not differ significantly between the two diabetes groups. However, C-peptide levels were higher in the T2D group compared to the T1D group (**Table 1**). BMI values were consistent with the expected clinical profiles of each condition.

**Table 1:** Demographic and glycaemic characteristics of the patients. Data are presented as median [25th; 75th percentile].

Parameter	Healthy donors (n = 24)	T1D (n = 23)	T2D (n = 14)	*P*-value¹
Sex – male, n (%) *	9 (34%)	13 (57%)	11 (79%)	0.048^0^
Age, years *	27.5 [24; 37]	31 [21; 35]	39.5 [34.5; 52]	< 0.001¹
BMI, kg/m² *	23 [20.3; 26]	22 [19.7; 24]	31 [29; 32]	< 0.001¹
Disease duration, months	Not applicable	6 [2; 9]	4.5 [2; 14]	0.885
Fasting glucose, mmol/L	4.6 [4.3; 5.1]	6.7 [5.9; 10]	7 [5.1; 8]	0.654
HbA1c, %	5.1 [5; 5.3]	7.4 [6.1; 9.3]	6.95 [6.35; 8.15]	0.467
C-peptide, ng/mL *	1.8 [1.3; 2.4]	1.02 [0.83; 1.56]	4.1 [2.7; 4.9]	< 0.001¹
MCP-1, pg/ml	182.2 [149.5; 203.2]	212.4 [147.1; 262.3]	240.7 [162.3; 302.8]	0.536
GADA, U/mL	0.32 [0.1; 1.04]	104.6 [15.05; 280.24]	0.35 [0.1; 1.32]	< 0.0000001^1^
IA-2A, U/mL	1.16 [0.6; 4.24]	7.1 [1.19; 269.65]	1 [0.52; 1]	0.001^1^
ICA, U/mL	0.38 [0.29; 0.46]	0.28 [0.21; 0.43]	0.32 [0.25; 0.35]	0.17
IAA, U/mL	2.2 [1.46; 3.3]	3.65 [2.47; 4.86]	3.08 [2.23; 3.3]	0.06
ZnT8A, U/mL	6.32 [4.79; 15.5]	168.66 [5.71; 1871.52]	–	0.027^1^

^0^Chi-square test across all groups. All expected cell counts > 5. ¹Mann–Whitney U test between diabetes groups. *Statistically significant differences between groups.

Plasma glucose and C-peptide levels were measured using the automated biochemical analyzer Architect c8000 (Abbott Laboratories, USA). HbA1c was assessed according to National Glycohemoglobin Standardization Program (NGSP) criteria. Complete blood counts were performed using the Sysmex XN-1000 hematology analyzer (Sysmex Corporation, Japan). Autoantibodies against glutamic acid decarboxylase (GAD), >10 positive, tyrosine phosphatase (IA-2), >10 positive, insulin, >10 positive, islet cells, >1 positive and zinc transporter 8 autoantibodies (ZnT8), >15 positive were detected using standard laboratory methods. MCP-1 level was assessed by enzyme-linked immunosorbent assay (Human MCP-1 ELISA kit, ThermoFisher, USA).

### Polychromatic flow cytometry of NK cells

Peripheral blood processing, sample preparation for flow cytometry, and panels of specific antibodies are described in details in **Supplementary Materials**.

Sample acquisition was performed using a BD LSRFortessa flow cytometer (BD Biosciences, USA), equipped with five lasers (355, 405, 488, 561, and 640 nm). Data acquisition was performed with BD FACSDiva software, while offline compensation correction and data analysis were carried out using FlowJo software (version 10.0; BD, San Diego, CA, USA). Gating strategies for all three panels are provided in the Supplementary Materials (**Supplementary Figures 1–3**).

### Statistical analysis

Statistical analyses were conducted using GraphPad Prism software (version 8.0; La Jolla, CA, USA). For variables with normal distribution, comparisons between groups were performed using Student’s t-test. For non-normally distributed data, the Mann–Whitney U test or the Kruskal–Wallis test was applied for comparisons between two or more groups, respectively. Differences were considered statistically significant at P < 0.05. All exact P-values are reported in the Supplementary Materials (**Supplementary Table S1**). Spearman correlation coefficients were used to assess pairwise correlations between the variables. The sample sizes of the three subgroups are relatively low (n = 24 for HD, n = 23 for T1D, n = 14 for T2D). This implies that at the significance level of 0.05, only the following Spearman correlations r could be targeted for detection with an adequate statistical power of 0.8: |r| > 0.55 for HD and T1D and |r| > 0.7 for T2D. For the same significance level and statistical power, the Student’s t and the Mann–Whitney U two-sample tests are sensitive for detecting only the following effect size (Cohen’s d value): |d| > 0.85 for comparing HD vs T1D and |d| > 0.95 for comparisons with T2D group. The power analysis above does not take into account the presence of missing data for some variables (up to 1.64% for iNKT cells, 4.92% for EOMES+ NK cells, 11.5% for NK cells expressing granzyme B and perforin).

To test the role of the age variable in explaining the differences in intergroup means, we built linear regressions [variable] = β_0_ + β_1_·[group] + β_2_·[age], where [variable] and [age] are the vectors of values of the variable of interest and of the age variable pulled from two groups, and [group] is the binary indicator with the elements equal to 1 (group 1) and 0 (group 2) in corresponding positions. If β_2_ is set to zero, testing for significance that β_1_ ≠ 0 is equivalent to conducting a Student’s t-test for the difference in means between two groups. Therefore, age was considered to be a confounding variable if β_2_ differed from zero with statistical significance. The graphical representation of the statistically significant correlations (at p < 0.05) in the form of the graph networks and the heatmaps was performed using the R packages igraph (version 1.6.0) and ggplot2 (version 3.5.2).

## Results

We first assessed the distribution of major lymphocyte subsets (T cells, B cells and NK cells) across the studied cohorts based on the flow cytometry data.

### Reduced NK cell population in patients with type 1 diabetes

**Figure 1:**
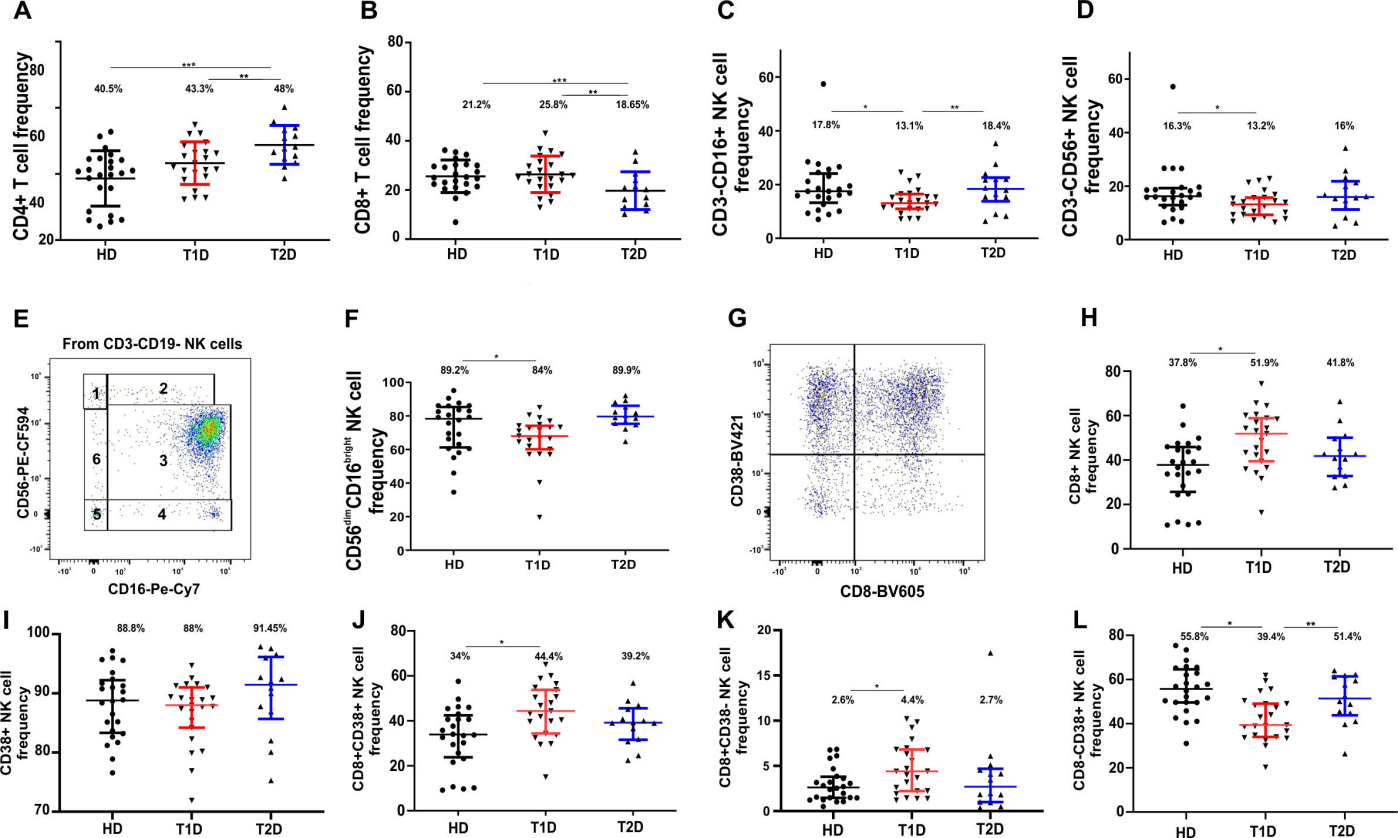
Frequency of CD4+, CD8+ T cells, and NK cell subsets differentially expressing CD16, CD56, CD8, and CD38 in peripheral blood of healthy donors and patients with T1D and T2D. **(A)** Proportion of CD4+ T cells. **(B)** Proportion of CD8+ T cells. **(C)** Proportion of CD3-CD16+ NK cells. **(D)** Proportion of CD3-CD56+ NK cells. **(E)** Representative flow cytometry CD16/CD56 dot plot showing the distribution of CD3-CD19- NK cells isolated from all CD45+ lymphocytes with the gates of six NK cell subtypes based on CD16 and CD56 expression. **(F)** Proportion of the effector CD56^dim^CD16^bright^ subtype 3 NK cells. A4; **(G)** Representative CD8/CD38 dot plot of the patient with T1D showing all CD45+CD3-CD19- NK cells. **(H)** Proportion of total CD8+ NK cells. **(I)** Proportion of total CD38+ NK cells. **(J)** Proportion of CD8+CD38+ NK cells. **(K)** Proportion of CD8+CD38- NK cells. **(L)** Proportion of CD8-CD38+ NK cells across the three study groups. Data are presented as medians with interquartile ranges. The values above data distributions correspond to median values. A single asterisk (*) denotes statistically significant differences at p < 0.05 between healthy donors and T1D; a double asterisk (**) indicates significant differences between T1D and T2D; a triple asterisk (***) indicates significant differences between HD and T2D. A patient with T1D was used as a case study in the dot plots.

No significant differences were observed in the frequencies of T and B lymphocytes between groups (**Table 2**). However, in the T2D group, a significant decrease in CD8+ T cells (P = 0.0148) and an increase in CD4+ T cells (P = 0.0002) were detected (**Figures 1A, B**). Patients with T1D exhibited a reduction in CD3-CD16+ and CD3-CD56+ NK cell subsets (P = 0.019 and 0.0227, respectively) compared to HD (**Figures 1C, D**, **Table 2**).

**Table 2:** Frequencies of major lymphocyte populations including NK cells expressing CD16, CD56, CD8, and CD38 in peripheral blood of healthy donors and patients with T1D and T2D.

Immune population	Healthy donors (HD), %	T1D, %	T2D, %
CD19+ CD3- B cells	9.89 [7.85-11.58]	10.2 [8.6- 13.6]	11.1 [10.05-12.8]
CD19- CD3+ T cells	72.2 [69.03-75.53]	72.9 [68.70-78.50]	71.05 [64.48-75.85]
CD3+CD8+ T cells	25.15 [21.65-30.15]	25.80 [21.50-31.40]	**18.65 [12.98-24.68] ♱, ⤉**
CD3+CD4+ T cells	40.50 [30.38-44.43]	43.30 [39.20-48.60]	**48.00 [44.38-60.30]♱♱♱,⤉**
CD3-CD19- NK cells	17.7 [13.23-19.9]	14.2 [10.8-17.00]	16.7 [12.5-22.45]
CD3-CD16+ NK cells	17.75 [13.28-24.15]	13.1 [11.8- 16.50]*, ⤉	18.4 [13.81-22.58]
CD3-CD56+ NK cells	16.25 [12.9-19.3]	13.2 [9.3- 15.7]*, ⤉	15.95 [11.29-21.85]
CD56brightCD16- Subtype 1	1.06 [0.23-2.30]	1.26 [0.60-1.84]	0.92 [0.44-1.53]
CD56brightCD16+/++ Subtype 2	2.29 [1.52-3.5]	2.38 [1.53-5.33]	2.40 [1.51-3.33]
CD56dimCD16bright Subtype 3	89.2 [80.63-92.7]	84.00 [80.10-87.10]*,⤉⤉⤉	**89.85 [87.70-93.05], ⤉⤉⤉**
CD56-CD16++ Subtype 4	2.74 [1.59-5.63]	4.29 [2.68-6.26]	2.44 [1.85-5.00]
CD56-CD16- Subtype 5	2.76 [1.43-5.89]	**3.56 [2.66-8.17], ⤉⤉⤉**	**1.85 [1.27-2.22], ⤉⤉⤉**
CD56dimCD16- Subtype 6	0.93 [0.73-1.42]	**1.26 [0.67-1.76], ⤉**	**0.62 [0.31-0.95], ⤉**
CD8+CD38+ NK cells	33.95 [23.83-42.50]	44.40 [34.40-53.80], **	39.20 [31.63-45.63]
CD8-CD38+ NK cells	55.75 [49.63-64.63]	39.40 [34.00-49.10], ***, ⤉	**51.40 [43.85-61.45], ⤉**
CD8+CD38- NK cells	2.63 [1.48-3.80]	4.41 [2.22-6.82], *	2.71 [1.01-4.70]
CD8-CD38- NK cells	7.48 [4.15-13.13]	7.86 [5.36-9.11]	5.47 [2.86-9.36]
all CD8+ NK cells	37.83 [25.68-45.90]	51.90 [39.47-58.91], ***	41.80 [32.85-50.08]
all CD38+ NK cells	88.80 [83.33-92.23]	88.00 [84.20-91.00]	91.45 [85.68-96.15]

Statistically significant differences are marked in bold; *p < 0.05, **p < 0.01, ***p < 0.001 for HD vs T1D; ♱p < 0.05, ♱♱p < 0.01, ♱♱♱p < 0.001 for HD vs T2D; ⤉p < 0.05, ⤉⤉p < 0.01, ⤉⤉⤉p < 0.001 for T1D vs T2D.

### Reduction of effector CD56^dim^CD16^bright^ NK cells in patients with type 1 diabetes

Circulating peripheral blood NK cells represent a heterogeneous lymphocyte population, differing in their maturation status, migratory properties, and effector potential. The expression of CD56 and CD16 is acquired peripherally during the final stages of NK cell development and differentiation. These markers delineate a trajectory from the less mature, regulatory CD56^bright^CD16– subset through CD56^bright^CD16^dim^ cells, towards the terminally differentiated, highly cytotoxic effector CD56^dim^CD16^bright^ NK cells (41). Various gating strategies have been employed to define NK subpopulations based on CD16 and CD56 expression, ranging from four to seven distinct subsets, occasionally defining populations such as CD56^dim^CD16–, CD56^dim^CD16^dim^, and CD56–CD16^bright^ NK cells (42, 43). In our study, we classified six NK subpopulations, hereafter referred to as subtypes 1 to 6: CD56^bright^CD16^-^ NK subtype 1, CD56^bright^CD16^+/++^ NK subtype 2, CD56^dim^CD16^bright^ NK subtype 3, CD56^-^CD16^+/++^ NK subtype 4, CD56^-^CD16^-^ NK, subtype 5, CD56^dim^CD16^-^ NK, subtype 6 (**Figure 1E**).

In patients with T1D, we observed a significant reduction (p = 0.0489) in the dominant effector NK cell subset CD56^dim^CD16^bright^ (Subtype 3), as shown in **Figure 1F**, **Table 2**. This decline occurred alongside a relative increase in other NK subpopulations; however, these changes did not reach statistical significance across the study groups (**Table 2**).

### Increased frequency of NK8+ and CD8^+^CD38^+^ NK cell subpopulation in patients with T1D

Special attention is warranted for NK cell co-receptors such as CD8 and CD38, whose roles on NK cells may be multifaceted. The association between the glycoprotein CD38 and CD16 is important for the acquisition of an effector cytotoxic phenotype by NK cells (44). In activated NK cells, CD38 signaling induces a cytotoxic response with granzyme and cytokine release. The functions of NK cells expressing CD8α remain controversial. We compared the frequencies of NK cells expressing CD8 (defined further as NK8+ cells) or CD38 or co-expressing both (**Figures 1G-I**). Assessing CD8 and CD38 expression on NK cells independently, it was an increase in CD8 expression (р=0.0024) that was noted in T1D, while the frequency of CD38-expressing NK cells remained unchanged, **Figures 1H, I**, **Table 2**. In T1D, an increase was observed in the double-positive CD8^+^CD38^+^ NK cell subpopulation compared to healthy donors (p = 0.0045) (**Figure 1J**, **Table 2**), as well as in CD8^+^CD38^-^ NK cells (p = 0.0311) (**Figure 1K**, **Table 2**), along with a reduction in CD8^-^CD38^+^ NK cells (p = 0.0045) (**Figure 1L**, **Table 2**).

It has been shown that NK cells with a CD56^bright^CD16^-^CD38^+^ phenotype may participate in adenosine (ADO) production, which inhibits autologous CD4 T cell proliferation (45). Therefore, we investigated the frequency of these cells in diabetes, examining all CD56^bright^CD16^-^ NK subtype 1 cells on a CD8/CD38 dot plot. However, we did not observe significant differences in CD38 expression among total CD56^bright^CD16^-^ NK subtype 1 cells or their CD8^+^ and CD8^-^ subpopulations (**Supplementary Table S1**).

### Increased frequency of NK8+, CD8+CD161+, CD8+NKG2A+, and CD8+EOMES+ NK cell subpopulations in patients with T1D

**Figure 2:**
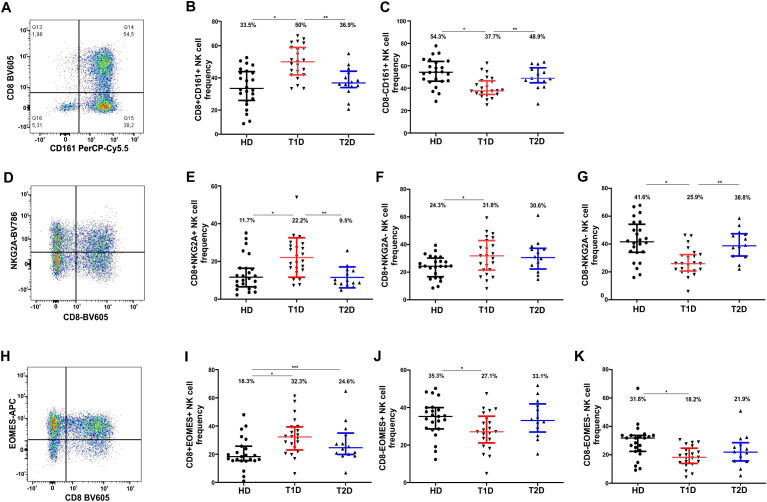
Changes in the frequency of NK cells and their subpopulations differentially expressing CD8, CD161, NKG2A, and EOMES in peripheral blood of healthy donors and patients with T1D and T2D. **(A)** Representative dot plot of CD161/CD8 showing the distribution of CD3-CD19- NK cells isolated from all CD45+ lymphocytes used to distinguish NK cell subtypes based on CD8 and CD161 expression. **(B)** Proportions of CD8+CD161+ NK cells. **(C)** CD8−CD161+ (NK cells across the three study groups. **(D)** B1 – Representative dot plot of CD8/NKG2A with CD3-CD19- NK cells isolated from all CD45+ lymphocytes used to distinguish NK cell subtypes based on CD8 and NKG2A expression; **(E-G)** Proportions of CD8+NKG2A+ **(E)**, CD8+NKG2A− **(F)**, and CD8−NKG2A− **(G)** NK cells across the three study groups; **(H)** –D Representative dot plot of CD8/EOMES used to distinguish NK cell subtypes based on CD8 and EOMES expression; **(I-J)** Proportions of CD8+EOMES+ **(I)**, CD8−EOMES+ **(J)**, and CD8−EOMES− **(K)** NK cells across the three study groups. Data are presented as medians with interquartile ranges. Numbers above data distributions indicate medians. Asterisk “*” denotes significant differences with p < 0.05 between healthy donors and T1D; “**” – significant differences between T1D and T2D; “***” – significant differences between healthy donors and T2D. A healthy donor was used as a case study in the dot plots.

The effector activity of NK cells is governed by the cumulative expression of inhibitory (e.g. NKG2A) and activating (e.g. NKG2D, NKp46, LFA-1) receptors, allowing them to exert cytolytic effects not only on aberrantly functioning cells but also on immune cells, including DCs and activated CD4+ and CD8+ T cells. One of the key immunomodulatory mechanisms of NK cells that could restrain autoimmune inflammation is the regulation of immune cell activation and the limitation of immune response magnitude. Upon analysis of all NK cells on a CD8/CD161 dot plot (**Figure 2A**) across three studied groups, we identified a significant increase in the frequency of CD8+CD161+ NK cells in T1D group compared to HD (p < 0.0001) and to T2D group (p = 0.0012) (**Figure 2B**, **Table 3**). It was accompanied by a significant reduction in the proportion of CD8−CD161+ NK cells against both HD (p = 0.0002) and T2D (p = 0.0055) groups (**Figure 2C**, **Table 3**). No changes was observed between T2D and HD. When evaluating CD8 and CD161 expression on NK cells independently, we again observed a statistically significant increase in CD8 expression (p < 0.0001), but not in CD161 (**Table 3**), in patients with T1D.

**Table 3:** Frequency of NK cell subpopulations expressing CD8, CD161, NKG2A, and EOMES in the peripheral blood of healthy donors and patients with T1D and T2D.

Immune population	Healthy donors (HD), %	TID, %	T2D, %
CD8+CD161+ NK cells	33.50 [26.05-43.95]	50.00 [41.90-58.90]***, ⤉⤉	**36.85 [34.05-44.18], ⤉⤉**
CD8-CD161+ NK cells	54.30 [46.33-63.95]	37.70 [34.50-46.60]***, ⤉⤉	**48.85 [44.90-58.28], ⤉⤉**
CD8+CD161- NK cells	2.22 [0.68-5.52]	2.94 [0.96-5.99]	4.10 [1.56-6.99]
CD8-CD161- NK cells	6.08 [2.66-10.06]	4.79 [1.86-7.93]	4.93 [3.48-11.18]
all CD8+ NK cells	38.04 [27.53-47.41]	54.39 [47.54-62.71]***, ⤉⤉	**41.53 [34.66-49.56], ⤉⤉**
all CD161+ NK cells	89.85 [85.10-96.20]	90.80 [86.70-97.30]	87.80 [80.93-94.78]
CD8+NKG2A+ NK cells	11.65 [6.58-16.45]	20.20 [15.40-28.10]**, ⤉⤉⤉	**9.50 [8.10-15.28], ⤉⤉⤉**
CD8-NKG2A+ NK cells	18.90 [14.80-24.55]	18.20 [13.30-25.30]	16.25 [10.88-22.08]
CD8+NKG2A- NK cells	24.30 [16.68-30.20]	31.80 [21.50-42.90]*	30.55 [22.33-37.33]
CD8-NKG2A- NK cells	41.60 [34.10-54.20]	25.90 [20.60-32.40]***, ⤉⤉	**38.75 [31.45-47.45],⤉⤉**
all NKG2A+ NK cells	29.20 [23.13-42.40]	38.20 [27.65-52.50]	**25.30 [18.81-37.35], ⤉**
CD8+EOMES+ NK cells	18.30 [15.40-25.70]	32.30 [23.05-39.40], ***	**24.60 [19.80-35.05], ♱**
CD8-EOMES+ NK cells	35.30 [28.60-40.00]	27.10 [21.20-35.40], *	33.10 [26.98-41.95]
CD8+EOMES- NK cells	14.90 [10.30-19.80]	16.60 [11.40-28.55]	14.05 [9.41-19.78]
CD8-EOMES- NK cells	31.80 [22.40-33.70]	18.20 [13.95-24.70], ***	21.90 [15.70-28.55]
all EOMES+ NK cells	58.62 [48.63-64.52]	60.51 [48.59-74.83]	65.55 [48.34-72.37]

Statistically significant differences are highlighted in bold; *p < 0.05, **p < 0.01, ***p < 0.001 for comparisons between HD and T1D; ♱p < 0.05, ♱♱p < 0.01 for comparisons between HD and T2D; ↑p < 0.05, ↑↑p < 0.01, ↑↑↑p < 0.001 for comparisons between T1D and T2D.

The inhibitory receptor NKG2A plays an essential role in modulating the lytic capacity of NK cells by attenuating their effector function (46). We observed a significant increase in the frequency of CD8+NKG2A+ NK cells in T1D group compared to HD (p < 0.0028) and to T2D group (p = 0.0004) (**Figure 2E**, **Table 3**), alongside an increase in CD8+NKG2A− NK cells, and an almost twofold reduction in the proportion of double-negative CD8−NKG2A− NK cells (**Figures 2F, G**, **Table 3**). At the same time, we observed no differences between HD and T2D group. Notably, we did not detect any changes in the frequency of NK cells co-expressing CD161 and NKG2A. Similarly, no differences were observed when assessing NKG2A expression on NK cells independently of CD8 (**Supplementary Table S1**). Although the proportion of NKG2A-positive NK cells increased by 30% in the T1D group, this change did not reach statistical significance (p = 0.1185, **Supplementary Table S1**).

Expression of the transcription factor EOMES is critical for the proper maturation and functional maintenance of NK cells, particularly in relation to their cytotoxicity mediated by the release of lytic granules such as perforin and granzymes (47). We observed an increased frequency of double-positive CD8+EOMES+ NK cells in the T1D group (p = 0.0008) and in the T2D group (p = 0.0383) compared to HD (**Table 3**, **Figures 2H, I**). In T1D, we also noted a reduction in the proportion of CD8-negative NK cells expressing EOMES compared to healthy donors (p = 0.0261), as well as a decrease in double-negative CD8−EOMES− NK cells (p = 0.0009) (**Table 3**, **Figures 2J, K**). However, the total number of EOMES-positive NK cells did not differ significantly across the patient groups (**Table 1**).

We also assessed the frequency of circulating CD161+NKG2A+ (**Supplementary Figure 2E**), CD161+EOMES+ (**Supplementary Figure 2F**), NKG2A+EOMES+ (**Supplementary Figure 2H**) NK cell subsets but did not observe any significant differences between the study groups (**Supplementary Table 1**).

### Cytotoxic potential of NK cells

**Figure 3:**
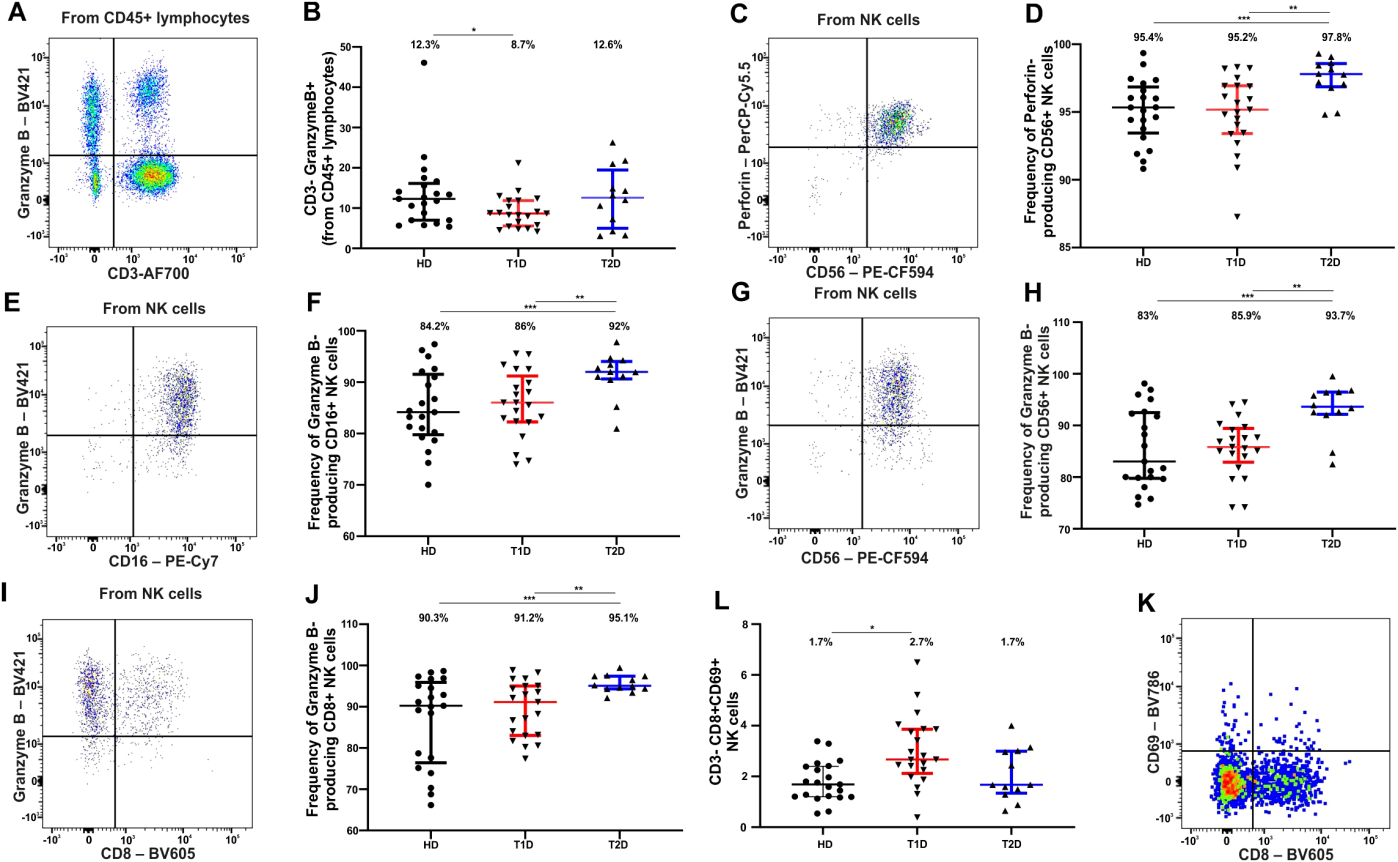
Cytotoxic potential of NK cells, determined by the presence of granules containing perforin and granzyme B, in peripheral blood of healthy donors and patients with T1D and T2D. **(A)** Representative dot plot CD3-AF700/Granzyme B-BV421 (used to assess granzyme B-expressing CD3- cells among all CD45+ lymphocytes. **(B)** Frequency of CD3- Granzyme B+ cells among the three study groups. **(C)** Representative dot plot CD56/Perforin showing all NK cells, used to assess the proportion of Perforin+ and Perforin- CD56+ NK cells to calculate the perforin potential of CD56+ NK cells. **(D)** Perforin potential of CD56+ NK cells among the three study groups. **(E)** Representative dot plot CD16/Granzyme B showing all NK cells, used to assess the proportion of Granzyme B+ and Granzyme B- CD16+ NK cells to calculate the granzyme potential of CD16+ NK cells. **(F)** B4 – Granzyme potential of CD16+ NK cells. **(G)** Representative dot plot CD56/Granzyme B showing all NK cells, used to assess the proportion of Granzyme B+ and Granzyme B- CD56+ NK cells to calculate the granzyme potential of CD56+ NK cells. **(H)** Granzyme potential of CD56+ NK cells. **(I)** Dot plot CD8/Granzyme B showing all NK cells, used to assess the proportion of Granzyme B+ and Granzyme B- CD8+ NK cells to calculate the granzyme potential of NK8+ cells **(J)**. **(K)** Representative dot plot CD8-BV605/CD69-BV786 used to evaluate the frequency of CD8+CD69+ NK cells among all NK cells. **(L)** Frequency of CD8+CD69+ NK cells across the study groups. Data are presented as medians with interquartile ranges. Numbers above data distributions indicate medians. Asterisk “*” denotes significant differences with p < 0.05 between healthy donors and T1D; “**” – significant differences between T1D and T2D; “***” – significant differences between healthy donors and T2D. A healthy donor was used as a case study in the representative dot plots.

Cytotoxicity mediated by NK cells, either via secretion of lytic granules containing perforin and granzymes or through induction of apoptosis in target cells, is one of the key functional properties of NK cells. We assessed the cytotoxic potential of NK cells by evaluating the presence of granules containing perforin and granzyme B. Upon quantifying the proportion of granzyme B-positive among lineage-negative CD3− lymphocytes (**Figure 3A**), we observed a decrease in their frequency in TID compared to the control group (p = 0.033, **Figure 3B**, **Table 4**). No differences were found in the frequency of perforin-containing granules in CD3− cells across the study groups. Analysis of the proportion of perforin- and granzyme B-positive granules and the evaluation of perforin and granzyme potential in CD16+ and CD56+ NK cells revealed an increased perforin potential in CD56+ NK cells in T2D compared to both healthy donors (p = 0.0031) and T1D (p = 0.0031), **Table 4**, **Figures 3C, D**. Granzyme potential was also elevated in T2D in both CD16+ (**Table 4**, **Figures 3E, F**) and CD56+ NK cells (**Table 4**, **Figures 3G, H**). Interestingly, among NK8+ cells, which increase in frequency in T1D and remain at control levels in T2D, granzyme potential was elevated in T2D but not in T1D (**Table 4**, **Figures 3I, J**). However, the proportion of activated cells within NK8+ (as indicated by CD69 expression) was higher in T1D (**Figures 3K, L**, **Table 4**).

**Table 4:** Frequency of NK cells expressing granzyme В and perforin in peripheral blood of healthy donors and patients with T1D and T2D.

Immune population	Healthy donors (HD), %	TID, %	T2D, %
CD3-Perforin+	11.8 [6.85-16.55]	10.1 [6.64-11.95]	14.85 [11.45-21.68]
CD3-Granzyme B+	12.3 [7.03-16.15]	8.7 [5.615-11.90], *	12.6 [5.010-19.48]
Perforin potential of CD16+NK cells	94.23 [91.72-95.57]	93.68 [92.95-95.7]	95.65 [93.88-96.07]
Perforin potential of CD56+NK cells	95.35 [93.45-96.86]	95.19 [93.42-96.94]	**97.8 [96.87-98.59], ♱♱, ⤉⤉**
Perforin potential of CD8+NKcells	98.5 [95.94-99.23]	98.35 [95.89-99.22]	98.74 [98.33-99.08]
Granzyme B potential of CD16+NK cells	84.2 [79.79-91.56]	86.03 [82.27-91.22]	**92 [90.64-94.06], ♱, ⤉**
Granzyme B potential of CD56+NK cells	83.06 [79.78-92.50]	85.86 [82.93-89.43]	**93.66 [92.17-96.48], ♱♱, ⤉⤉**
Perforin+GranzymeB+NK cells	86.1 [77.55-90.7]	87.6 [83.95-90.75]	**90.95 [89.13-93.13], ♱, ⤉**
Perforin potential of CD8+NK cells	98.5 [95.94-99.23]	98.35 [95.89-99.22]	98.74 [98.33-99.08]
GranzymeB potential of CD8+NK cells	90.26 [76.43-95.94]	91.16 [83.08-95.07]	**95.11 [94.37-97.44], ♱, ⤉⤉**
CD3-CD69+	2.06 [1.195-3.25]	2.44 [1.38-3.78]	2.72 [1.823-7.015]
CD3+CD69+	69.9 [64-73.15]	68.1 [61.65-72.85]	62.8 [57.95-71.25]
CD3-CD8+CD69+NK	1.69 [1.205-2.405]	2.67 [2.120-3.865], ***	1.68 [1.343-3]

Statistically significant differences are shown in bold. *p<0.05, **p<0.01, ***p<0.001 when comparing HD and T1D; ♱p<0.05, ♱♱p<0.01, ♱♱♱p<0.001 when comparing HD and T2D; ⤉p<0.05, ⤉⤉p<0.01, ⤉⤉⤉p<0.001 when comparing T1D and T2D.

### Frequency of NKT cells and their subpopulations in peripheral blood of patients with T1D, T2D, and healthy donors

**Figure 4:**
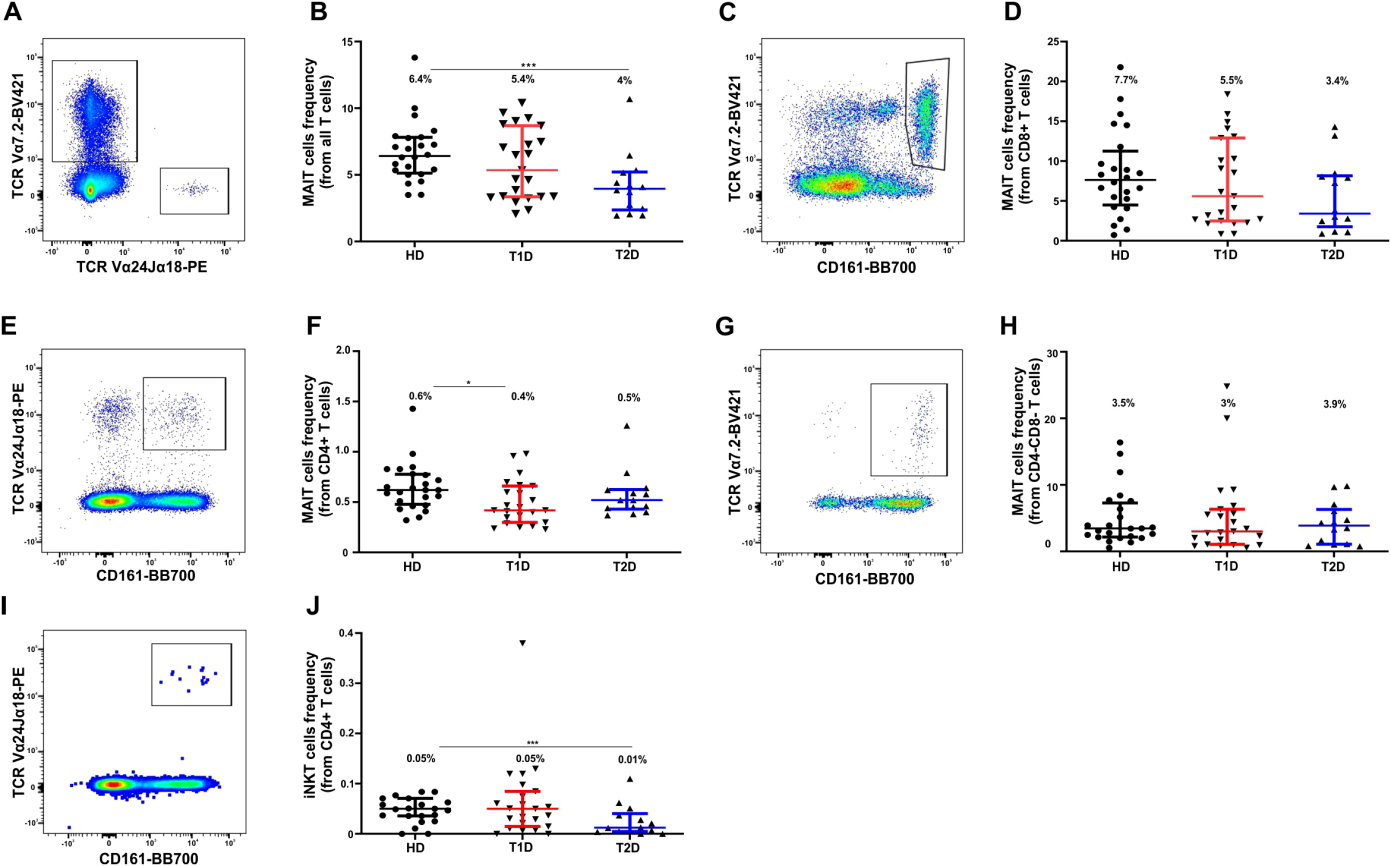
Changes in the frequency of NKT cells and their subpopulations (MAIT and iNKT) in peripheral blood of healthy donors and patients with type 1 and type 2 diabetes. **(A)** Analysis of total MAIT and iNKT lymphocyte populations among all CD3+ T cells based on expression of TCR Vα7.2 and TCR Vα24-Jα18, respectively, on TCR Vα7.2-BV421/TCR Vα24-Jα18-PE dot plot. **(B)** Proportion of total MAIT cells among CD3+ T cells in the three studied groups. **(C)** Dot plot CD161-BB700/TCR Vα7.2-BV421 showing distribution of cytotoxic CD8+ T cells and used to identify MAIT cells (as double-positive CD161+TCR Vα7.2+). **(D)** Frequency of CD8+MAIT cells among three study groups. **(E)** Dot plot CD161-BB700/TCR Vα7.2-BV421 with CD4+ T cells. **(F)** Frequency of CD4+MAIT cells among three study groups. **(G)** Dot plot CD161-BB700/TCR Vα7.2-BV421 with CD4-CD8- DN T cells **(H)** Frequency of DN MAIT cells among three study groups. **(I)** Dot plot CD161-BB700/TCR Vα24-Jα18-PE used to identify iNKT cells (as double-positive CD161+TCR Vα24-Jα18+) among CD4+ T cells **(J)**. Data are presented as medians with interquartile ranges. Numbers above data distributions indicate medians. Asterisk “*” denotes significant differences with p < 0.05 between healthy donors and T1D; “**” – significant differences between T1D and T2D; “***” – significant differences between healthy donors and T2D. A healthy donor was used as a case study in the representative dot plots.

NKT cells represent a distinct subset of αβ T lymphocytes. They form a heterogeneous group classified into three subpopulations depending on their antigen specificity and TCR expression: iNKT, type II NKT, and NKT-like cells including MAIT cells (48). Among all T cells, MAIT and iNKT cells were identified based on the expression of the TCR Vα7.2 and Vα24-Jα18 chains, respectively (; **Figure 4A**, **Supplementary Figure 2I**). In T2D, there was a significant reduction in MAIT cells compared with healthy donors (p = 0.0006, **Table 5**; **Figure 4B**). In T1D, only a non-significant trend towards MAIT cell reduction was observed (**Table 5**). The frequency of iNKT cells among all T cells did not differ between the study groups (**Table 5**).

We also assessed the frequency of MAIT cells by identifying them as double-positive TCR Vα7.2^+^CD161^++^ cells within CD4^+^, cytotoxic CD8^+^ and double-negative (DN) CD4^-^CD8^-^ T-cell subsets. We also assessed the frequency of MAIT cells within CD4^+^, cytotoxic CD8^+^ and double-negative (DN) CD4^-^CD8^-^ T-cell subsets by identifying them as double-positive TCR Vα7.2^+^CD161^++^ cells. The majority of MAIT cells were observed within CD8^+^ and DN T-cell subpopulations (**Table 5**). A trend towards more than twofold reduction of CD8^+^ MAIT cells was noted in T2D (p = 0.0765, **Figures 4C, D**, **Table 5**) compared with healthy donors. Interestingly, a significant reduction of CD4^+^ MAIT cells was observed in T1D (p = 0.0128) compared with the donor group (**Figures 4E, F**, **Table 5**). No differences were observed among DN MAIT cells (**Figures 4G, H**, **Table 5**).

**Table 5:** Frequency of NKT cell subpopulations, MAIT and iNKT cells in peripheral blood of healthy donors and patients with type 1 and type 2 diabetes mellitus.

Immune population	Healthy donors (HD), %	T1D, %	T2D, %
MAIT (from all CD3+ T cells)	6.42 [5.13-7.82]	5.36 [3.35-8.69]	**3.96 [2.36-5.21], ♱♱♱**
MAIT (from CD8+ T cells)	7.64 [4.49-11.27]	5.59 [2.49-12.90]	3.40 [1.76-8.16]
MAIT (from CD4+ T cells)	0.62 [0.48-0.78]	0.42 [0.30-0.66], *	0.52 [0.43-0.63]
MAIT (from DN T cells)	3.46 [2.17-7.28]	3.01[1.07-6.34]	3.90 [1.09-6.31]
iNKT (from all CD3+ T cells)	0.07 [0.04-0.18]	0.05 [0.03-0.16]	0.04 [0.02-0.11]
iNKT (from CD8+ T cells)	0.07 [0.03-0.18]	0.04 [0.01-0.08]	0.03 [0.00-0.11]
iNKT (from CD4+ T cells)	0.05 [0.04-0.07]	0.05 [0.02-0.09]	**0.01 [0.00-0.04], ♱♱**
iNKT (from DN T cells)	0.65 [0.20-1.77]	0.42 [0.09-0.83]	0.44 [0.20-1.87]

Statistically significant differences are shown in bold; *p<0.05, **p<0.01, ***p<0.001 when comparing HD and T1D; ♱p<0.05, ♱♱p<0.01, ♱♱♱p<0.001 when comparing HD and T2D; ⤉p<0.05, ⤉⤉p<0.01, ⤉⤉⤉p<0.001 when comparing T1D and T2D.

In the analysis of iNKT cells within CD8^+^ cytotoxic, CD4^+^, and CD4^-^CD8^-^ DN T-cell subsets — defined as CD161^+^TCR Vα24-Jα18^+^ double-positive cells (gating strategy in **Supplementary Figure 2M–O**) — and comprising less than 0.1% of all CD3^+^ T cells, we observed a general trend towards reduction in both T1D and T2D, reaching statistical significance for CD4^+^ iNKT cells in T2D compared with healthy controls (p = 0.0101, **Figures 4I, J**, **Table 5**, **Supplementary Table 1**). The analysis of general NKT cells defined by the immunophenotype CD3^+^CD16^+^ or CD3^+^CD56^+^ revealed no significant differences between the groups (**Supplementary Table 1**).

### Relationships between immune cell populations and metabolism of patients with T1D and T2D

**Figure 5:**
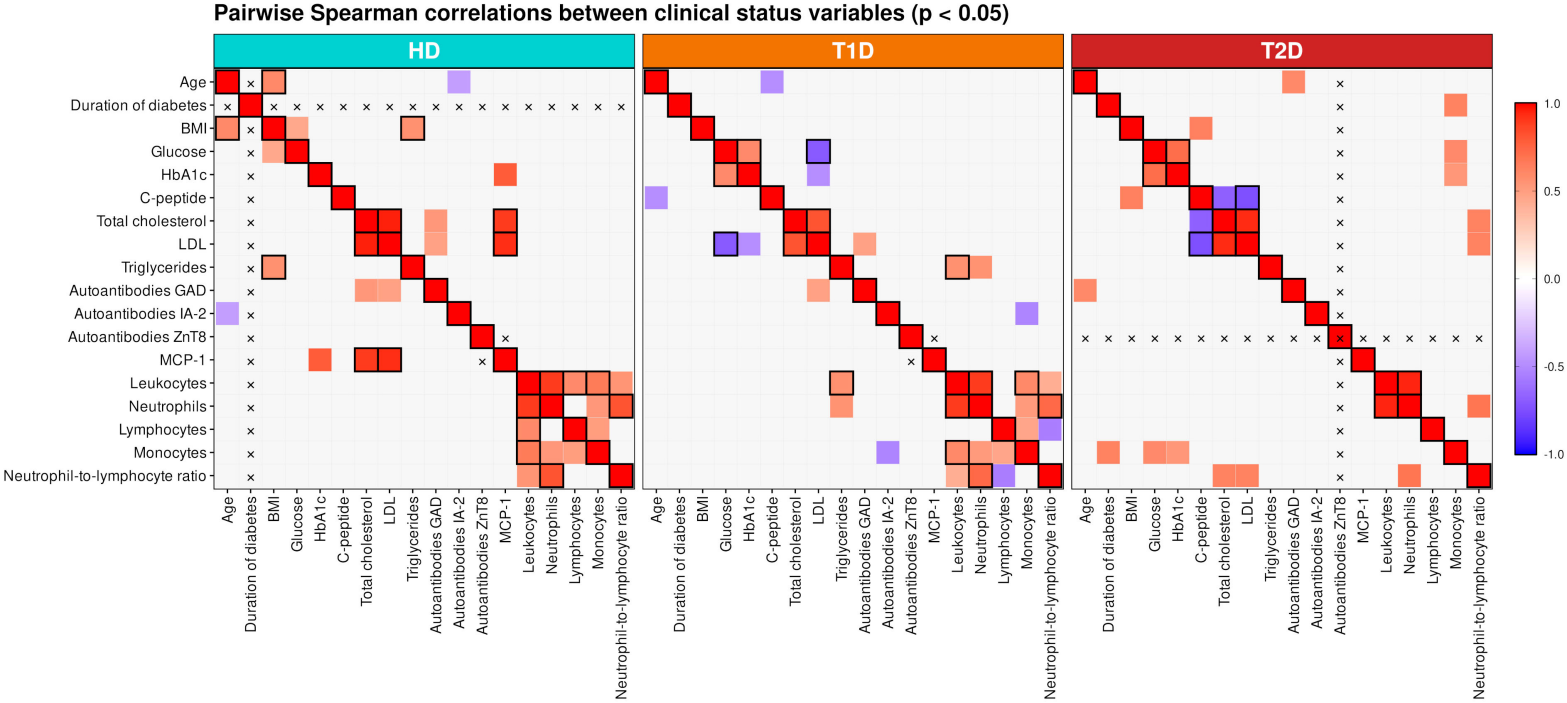
Heatmap of correlation relationships between clinical parameters in the study groups: age, duration of diabetes, BMI, glucose and HbA1c levels, C-peptide, total cholesterol, LDL and triglyceride levels, autoantibodies GAD, IA-2, ZnT8, absolute counts of leukocytes, neutrophils, lymphocytes, monocytes, and the neutrophil-to-lymphocyte ratio. Heatmap cells with statistically significant correlations at p < 0.05 are colored and the cells with significance level of p < 0.005 are additionally outlined with black borders. The cells with insignificant correlations (p < 0.05) are marked with gray color. The cells with missing or not applicable data are labeled with symbol "x".

Correlation analysis of the clinical parameters in T1D, presented as a heatmap (**Figure 5**), did not reveal statistically significant correlations between autoantibodies and C-peptide levels. However, C-peptide levels showed an inverse correlation with age, which was not observed in the control group. In T2D, we observed a positive correlation between C-peptide levels and BMI, and an inverse correlation with total cholesterol and LDL levels. Furthermore, in T2D, the number of monocytes positively correlated with glucose and HbA1c levels, supporting the notion of myelopoiesis activation during the meta-inflammation characteristic of T2D. Monocyte count also correlated with disease duration in T2D. The neutrophil-to-lymphocyte ratio, a recognized marker of metabolic dysfunction, correlated with both total cholesterol and LDL levels. In healthy individuals, we observed balanced inter-correlations between major leukocyte groups. Notably, BMI in this group positively correlated with triglyceride levels.

**Figure 6:**
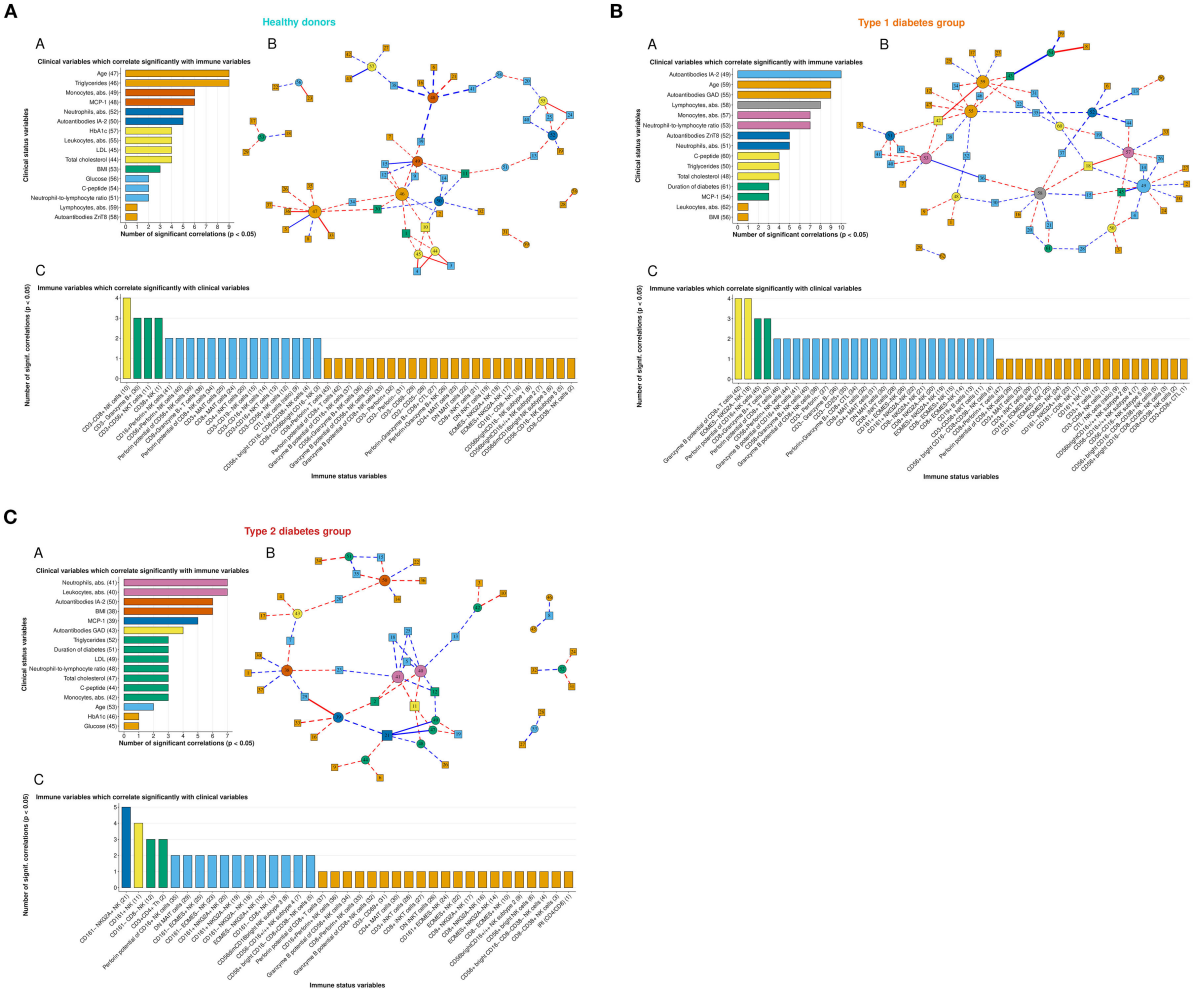
**(A)** Correlation network graph of clinical parameters and immune cell populations in the group of healthy donors. (A) Distribution of clinical parameters according to the number of statistically significant correlations with immune cell populations. (B) Graph representation: circle nodes denote clinical parameters, rectangle nodes - immune cell populations; red lines indicate positive correlations, blue lines - negative (inverse) correlations; line width is proportional to the absolute value of correlation coefficients; dashed lines indicate the significance level p<0.05, solid lines - p<0.005. (C) Distribution of immune cell populations according to the number of statistically significant correlations with clinical parameters. **(B)** Correlation network graph of clinical parameters and immune cell populations in the group of patients with T1D. (A) Distribution of clinical parameters according to the number of statistically significant correlations with immune cell populations. (B) Graph representation: circle nodes denote clinical parameters, rectangle nodes - immune cell populations; red lines indicate positive correlations, blue lines - negative (inverse) correlations; line width is proportional to the absolute value of correlation coefficients; dashed lines indicate the significance level p<0.05, solid lines - p<0.005. (C) Distribution of immune cell populations according to the number of statistically significant correlations with clinical parameters. **(C)** Correlation network graph of clinical parameters and immune cell populations in the group of patients with T2D. (A) Distribution of clinical parameters according to the number of statistically significant correlations with immune cell populations. (B) Graph representation: circle nodes denote clinical parameters, rectangle nodes - immune cell populations; red lines indicate positive correlations, blue lines - negative (inverse) correlations; line width is proportional to the absolute value of correlation coefficients; dashed lines indicate the significance level p<0.05, solid lines - p<0.005. (C) Distribution of immune cell populations according to the number of statistically significant correlations with clinical parameters.

Heatmaps of correlation matrices between peripheral blood immune cell populations and clinical parameters reflecting metabolic changes in patients with T1D and T2D are presented in **Supplementary Figures 4–6**. Visualization of correlation relationships in the form of graphs is shown in **Figures 6A–C**. Comparison of the graphs across the three study groups revealed that each group is characterized by specific clinical parameters (represented as graph nodes in circles) that demonstrate the highest number of connections (i.e. node degree) with immune cell populations (represented as squared nodes). Interestingly, in the T1D group, autoantibodies to GAD, IA-2, and ZnT8 emerged as major hubs – **Figure 6B**. In T1D, IA-2 autoantibody levels positively correlated with EOMES+, CD161–EOMES+, and CD8–EOMES+ NK cells, and negatively – with CD3–CD8+ NK lymphocytes, CD8+CD38–, CD56^bright^CD8+CD38– NK cell populations, as well as with EOMES-negative NK populations expressing CD8, CD161, or NKG2A, and with perforin potential of CD16+ NK cells. Notably, GAD autoantibody levels inversely correlated with CD8+ MAIT cells, granzyme potential of CD8+ NK cells, and activated CD3–CD25+ NK cells. Although T cells are not the main focus of this paper, it is worth noting that GAD autoantibodies positively correlated with CD3+ T lymphocytes, CD8+Granzyme B+ T cells, and perforin potential of CD8+ T cells. ZnT8 autoantibody levels negatively correlated with B cells and CD56–CD16– NK cells, possibly reflecting migration of these cells to the pancreas. In T1D, we observed no clear correlations between NK cells and their subsets with C-peptide levels, which reflect endogenous insulin production, except for the inverse correlations with EOMES–NKG2A– and CD161+NKG2A– NK cells, and a positive correlation with EOMES+NKG2A+ NK cells. We also observed a negative correlation between CD4+ MAIT cells and C-peptide, which may indicate CD4+ MAIT cell recruitment into the affected pancreas.

In T2D, the central nodes of the graph (based on node degree) with the greatest number of significant correlations were neutrophil and leukocyte counts, as well as BMI and MCP-1 levels (**Figure 6C**), reflecting the T2D-associated metaflammation. Leukocyte and neutrophil counts, as well as MCP-1 levels, correlated with the proportion of CD4+ T cells, highlighting the known involvement of both innate (neutrophils) and adaptive (CD4 Th cells) immune components in metaflammation. Leukocytes and neutrophils also correlated with the proportion of CD161+ NK cells, showing inverse correlations with CD161-negative and CD8+CD38–CD56^bright^ NK subsets, suggesting a proinflammatory role of CD161-positive NK cells in T2D. MCP-1 levels inversely correlated with CD161–NKG2A+ NK cells and positively correlated with CD8+NKG2A– and CD8+Perforin+ NK cells, as well as with the frequency of DN MAIT cells (p<0.005), which in turn negatively correlated with BMI. BMI also positively correlated with the proportion of CD161–EOMES– NK cells and inversely correlated with IRI and the perforin potential of CD8+ T cells. The main cytotoxic subset of NK cells – CD56^dim^CD16^bright^ (Subtype 3) – inversely correlated with glucose and HbA1c levels, possibly indicating migration of this NK population to inflamed tissues. Interestingly, C-peptide levels correlated with the frequency of all CD56^bright^ and CD161–NKG2A+ NK cells, again supporting a proinflammatory role of CD161+ NK cells in metaflammation associated with T2D.

Interestingly, we have noted that the correlation heatmap of immune parameters of HD group appears to be much denser that in T1D and T2D groups (data not shown). It supports the importance of the many balanced correlations for normal operation of any system. In both T1D and T2D, an inverse correlation was observed between the frequency of NK cells (both total and CD3-CD16+/CD3-CD56+ subsets) and cytotoxic CD8+ T cells (**Supplementary Figures 7A, B**).

Additionally, we checked if the age variable contributes to explaining the differences in intergroup means which were deemed significant. Namely, for individuals with T1D the respective populations are (1) overall NK cells (2), CD56^dim^CD16^bright^ subpopulation (3), CD8-expressing CD3−CD19− NK cells (4), CD8+CD38+ (5), CD8+CD161+ (6), CD8+NKG2A+, and (7) CD8+EOMES+ NK subsets. For individuals with T2D the relevant populations are (1) NKG2A+ NK cells and (2) MAIT cell subset within CD3+ T cell population. We found that including the age variable as a covariate to the regression of the immune cell subset on the binary variable that represents the group membership is justified only for overall NK cells (CD3−CD16+ NK cells): p=0.031 for contribution of group membership and p=0.018 for contribution of age. Age was not relevant in explaining the differences in means of the other variables.

## Discussion

**Figure 7:**
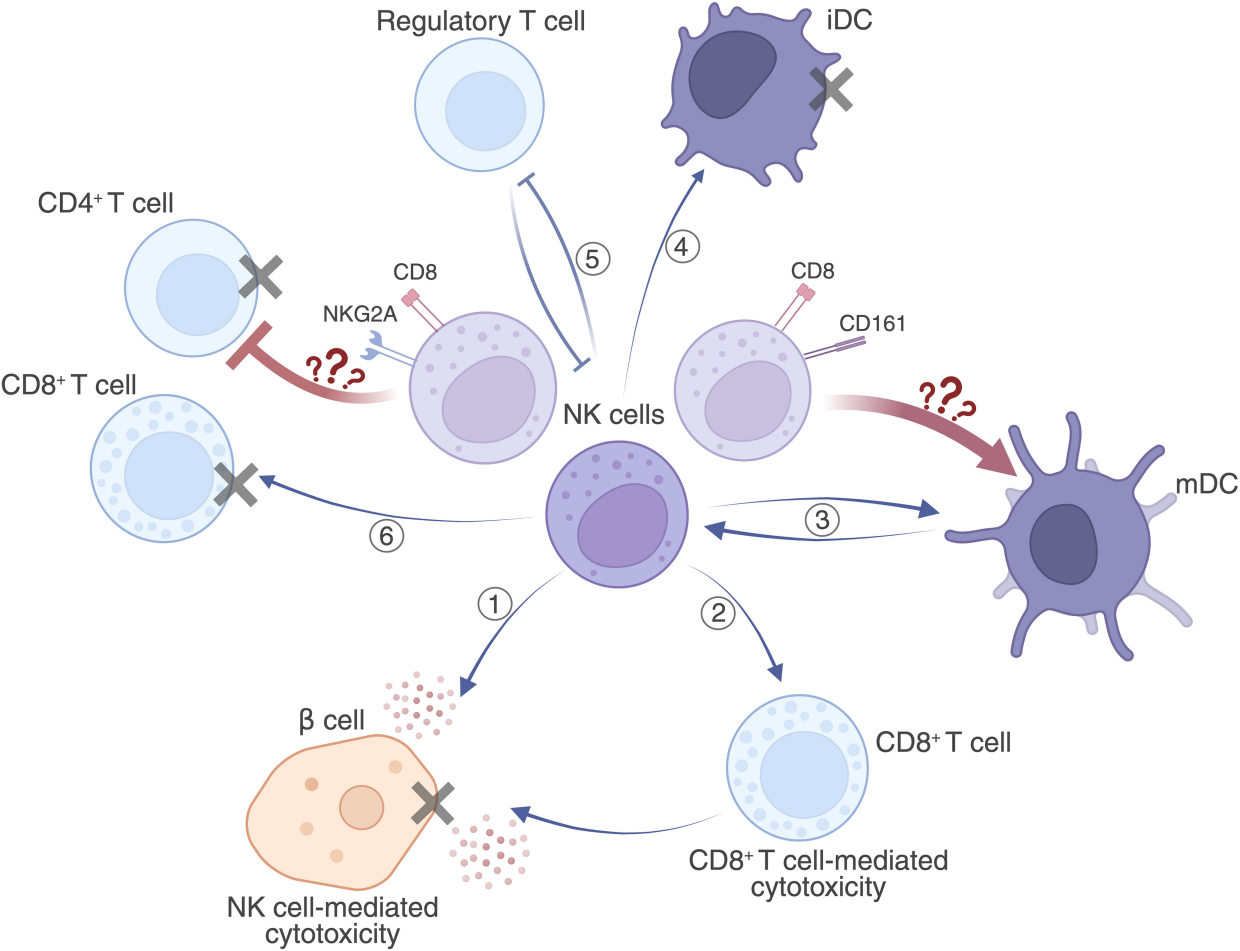
Possible pathways of NK cell involvement in the pathogenesis of pancreatic β-cells in T1D. 1. NK cells may exert cytotoxic effects on β-cells either through direct interaction via activating receptors with corresponding ligands on β-cells, or through the release of cytokines or other cytotoxic mediators; 2. NK cells may stimulate CD8^+^ T cells to expand and enhance their cytotoxic action against β-cells; 3. NK cells may enhance the activation of DCs via contact-dependent or cytokine-mediated mechanisms, sustaining the immune response. Interaction of CD161, an inhibitory NK cell receptor, with its ligand on mDCs inhibits NK cell cytotoxic activity and protects mDCs from NK-mediated lysis; 4. NK cells can lyse immature non-activated DCs, thereby indirectly promoting the selection of diabetogenic mDCs and CD8^+^ T cells; 5. NK cells may destabilise the action of Tregs, which in turn may inhibit NK cell function; 6. NK cells may direct their cytolytic activity against activated CD4^+^ and CD8^+^ T cells, thereby indirectly protecting β-cells from CD8^+^ T cell-mediated destruction. Interaction of NKG2A, an inhibitory NK cell receptor, with HLA-E, which is upregulated on activated CD4^+^ T cells and serves as a ligand for NKG2A, blocks NK-mediated lysis of activated CD4^+^ T cells and supports the diabetogenic CD4^+^ T cell population. Created in https://BioRender.com.

In the present study, we analyzed the role of NK cells and their subpopulations in the pathogenesis of T1D and T2D. NK cells may contribute to the breakdown of peripheral tolerance, leading to the development of autoimmune diseases. Representing a highly heterogeneous group of cells with diverse functional capacities, they are a double-edged sword capable of modulating the immune response in both pro-inflammatory and anti-inflammatory directions (49, 50, 51). During the initiation phase of the immune response and the priming stage of autoimmunity, NK cells may exhibit cytolytic activity by targeting β-cells that express stress-induced ligands for NK cell receptors (**Figure 7**, Pathway 1). In parallel, in several antiviral and antitumor experimental models in both animals and humans, NK cells have been shown to polarize specific T cell responses (27). NK cells can influence the proliferation of autoreactive T and B lymphocytes, and stimulate CD8+ T cells to expand and enhance their cytotoxicity against β-cells (**Figure 7**, Pathway 2). Upon activation by mature DCs, NK cells can in turn enhance DC activation through contact-dependent mechanisms or via IFNγ/TNFα signaling (**Figure 7**, Pathway 3) (27). They may also eliminate immature DCs, thereby amplifying the magnitude of the immune response (**Figure 7**, Pathway 4) (18, 19). Additionally, their activity may be regulated by Tregs, which reduce the expression of NKG2D on NK cells via TGFβ (**Figure 7**, Pathway 5) (52). During the clonal expansion phase, NK cells may lyse activated CD4 and CD8 T cells (**Figure 7**, Pathway 6), recognizing ligands on T cells for NKG2D, NKG2A, NKp46 activating receptors (46).

The maintenance of immune homeostasis, in which NK cells play a key role by regulating and shaping the immune response, is essential to prevent the development of excessive or autoimmune inflammation. The contribution of NK cells to the pathogenesis of T1D (6,10–12 (53)) and T2D (33–35) has been demonstrated in numerous studies; however, the exact mechanisms of action and the role of specific NK cell subpopulations remain unclear.

In T1D, we observed a reduction in the overall number of NK cells (**Figures 1C, D**, **Table 2**), which is consistent with previous findings reported in adults (13, 24) and children with T1D (54). This decrease may be explained by the migration of NK cells into the pancreas, a phenomenon observed during the initiation phase of the autoimmune process and continuing at the onset of T1D. In murine models, early NK cell recruitment has been demonstrated during the initial stages of insulitis, with a cytotoxic role in β-cell destruction in T1D (12). We also found a decrease in CD56^dim^CD16^bright^ NK cells (subtype 3) in T1D, which likely reflects the migration of this subpopulation to the pancreas (**Figure 1F**, **Table 2**). Interestingly, CD16 can be shed by metalloproteases during NK cell activation (55). A similar reduction in this NK cell subset has been previously reported in children with T1D (54). We did not observe significant changes in the number of regulatory CD56^bright^CD16- and CD56^bright^CD16^dim^ NK cell populations (**Table 2**).

When analyzing the expression of co-receptors CD8 and CD38 on NK cells, we observed that CD38 is expressed on nearly 90% of NK cells under normal conditions, and this proportion remained unchanged in both types of diabetes. However, in T1D, there was a marked increase, almost 1.5-fold, in CD8 expression on NK cells (**Figure 1H**, **Table 2**). The functions of CD8α-expressing NK cells remain unclear and somewhat contradictory. Some studies report that this subpopulation is more cytotoxic (56), being resistant to activation-induced apoptosis and capable of multiple target cell lysis (57). Conversely, other data suggest that the CD8αα homodimer on CD3-CD8+ NK cells enhances binding of the inhibitory receptor KIR3DL1 to HLA-I on target cells, thereby reducing cytotoxicity (58). Moreover, an inverse correlation has been observed between CD3-CD8+ NK cells and relapses in patients with relapsing-remitting multiple sclerosis, attributed to the suppressive regulatory effect of these NK cells on CD4 T cells (59). Interestingly, a previous study also noted an increase in CD16+CD8+CXCR3+ NK cells in individuals with autoantibodies who subsequently developed T1D (39). In our study, we found that T1D was associated with an increase in both CD8+CD38+ and CD8+CD38- NK cells (**Figures 1J, K**, **Table 2**). At the same time we revealed no correlations between the frequency of any CD8+ NK cell subsets with clinical parameters. It is noteworthy that CD56^bright^CD16- CD38+ NK cells have been implicated in ADO production, potentially inhibiting the proliferation of autologous CD4 T cells (45). However, we did not observe any significant differences in CD38 expression among CD56^bright^CD16- NK subtype 1 cells or their CD8+ and CD8- subpopulations (**Supplementary Table S1**).

NK cells express a heterogeneous repertoire of receptors, which enables them to distinguish between healthy cells and those rendered dysfunctional by various forms of cellular stress. Their functional activity is governed by a finely tuned balance between surface-expressed activating and inhibitory receptors, with cellular responses emerging as the integrated result of these competing signals. Activating receptors, such as NKp46 and NKG2D, typically recognize ligands that are upregulated in response to cellular stress, oncogenic transformation, or pathogen infection. In contrast, inhibitory receptors, such as killer cell immunoglobulin-like receptors (iKIRs) and CD94/NKG2A heterodimer, act as sentinels of self by surveying the expression of MHC class I molecules. These molecules are constitutively expressed on the surface of healthy cells but are frequently downregulated in virally infected or malignant cells or, conversely, upregulated upon activation, as observed in monocytes, macrophages, and T lymphocytes (46). An imbalance in the expression or signaling of activating versus inhibitory receptors may lead to the direct cytotoxic targeting of pancreatic β-cells or, alternatively, to impaired immunological tolerance through effects on dendritic cells, CD4+ and CD8+ effector T cells.

CD161 is expressed across multiple immune cell types, including NK cells, NKT cells, and Th17 cells. It belongs to the C-type lectin-like receptor (CLR) family. NK cells expressing CD161 may exhibit an enhanced capacity for IFNγ production in response to IL-12 and IL-18 stimulation compared with their CD161-negative counterparts, and they are generally associated with a proinflammatory phenotype (60). Conversely, engagement of CD161 on NK cells with its physiological ligand, lectin-like transcript-1 (LLT1), has been shown to inhibit both NK cell cytotoxicity and cytokine secretion (38, 61). Notably, LLT1 is expressed by various APCs, including TLR-activated plasmacytoid dendritic cells (pDCs), monocyte-derived dendritic cells (moDCs), and B cells following stimulation via TLR9, surface immunoglobulins, or CD40 (38). In this context, LLT1 expression serves a protective function, shielding activated APCs from NK cell-mediated lysis. In our study, we observed a significant increase in the frequency of CD8+CD161+ NK cells in individuals with T1D, relative to both healthy donors and those with T2D (**Figure 2B**, **Table 3**). We hypothesize that an increased proportion of peripheral NK cells with the potential to spare activated dendritic and B cells from apoptosis may contribute to the amplification of a diabetogenic immune response. Interestingly, a recent study also reported elevated CD161 expression by NK cells in children who were positive for two autoantibodies and subsequently developed T1D (62).

NKG2A CD161, also belongs to the C-type lectin-like receptor CLR family. On NK cells, NKG2A functions as an inhibitory receptor. Its ligand is the non-classical MHC class Ib molecule HLA-E. Recognition of HLA-E on target cells transmits an inhibitory signal to NK cells, thereby preventing their activation against the recognized cell (41). HLA-E expression is generally low across most cell types (63). However, increased expression of HLA-E has been reported on pancreatic β-cells in patients with T1D, which is thought to confer protection against NK cell-mediated cytotoxicity (64). Nonetheless, given the cross-talk between NK cells and other immune populations, NK cells may contribute to β-cell destruction indirectly by modulating the activity of cytotoxic CD8+ and helper CD4+ T cells. Indeed, interaction between NKG2A-expressing NK cells and activated CD4+ T cells has been shown to protect the latter from NK-mediated lysis, thereby supporting their expansion and the development of immune memory (65). Therapeutic blockade of the NKG2A–HLA-E axis has emerged as a promising strategy in the treatment of autoimmune diseases (63). Given this context, we sought to assess the relative abundance of NKG2A-positive NK cell subpopulations with putative diabetogenic properties. Our analysis revealed a significant increase in CD8+NKG2A+ NK cells in the T1D group compared with both healthy controls and individuals with T2D (**Figure 2E**, **Table 3**).

EOMES, belonging to the T-box family, is expressed at early stages of NK cell development and is required for terminal maturation and acquisition of cytotoxic effector function (66). NK cells co-expressing EOMES and T-bet exhibit enhanced antibody-dependent cellular cytotoxicity (ADCC) (67). In T1D, we observed an increased proportion of CD8^+^EOMES^+^ NK cells, a decreased frequency of CD8^-^EOMES^+^ NK cells, and a reduction in CD8^-^EOMES^-^ double-negative NK cells compared to healthy donors (**Table 3**, **Figures 2I–K**). However, the total number of EOMES-positive NK cells did not differ significantly between patient groups (**Table 3**). Interestingly, the CD8^+^EOMES^+^ NK cell subset was also elevated in T2D (**Table 3**).

It is noteworthy that we did not observe any changes in the frequency of NK cells co-expressing CD161 and NKG2A, CD161 and EOMES, or NKG2A and EOMES (**Supplementary Table 1**). These findings suggest that T1D is associated with an increased expression of CD161+, NKG2A+, and EOMES+ specifically on CD8+ NK cells. We further assessed the overall proportion of CD8+ NK cells regardless of co-expression with other antigens and found a significant increase in this population among individuals with T1D compared with healthy donors (**Tables 2**, **3**). By contrast, when evaluating the frequencies of CD161+, EOMES+, and NKG2A+ NK cells independently of CD8 expression, we did not observe statistically significant differences - only a trend towards elevation (**Supplementary Table 1**). Thus, the expansion of CD8+ NK cells and, more specifically, CD8+CD161+, CD8+NKG2A+, and CD8+EOMES+ subsets in T1D may indicate a special role for these NK cell populations in the disease. It is plausible that in T1D, these NK subpopulations exert an immunoregulatory function — preventing apoptosis of autoreactive CD4+ T cells via NKG2A expression and shielding activated dendritic cells and B cells from lysis through CD161, while resisting AICD themselves, potentially due to the protective role of CD8. This hypothesis warrants further experimental validation.

The role of NK cells and their subsets in the regulation of metaflammation associated with T2D remains both contradictory and insufficiently defined (37). Early studies have reported an increased proportion of CD56^bright^CD16− NK cells in VAT in the context of obesity (37, 68), yet the precise contributions of obesity-associated CD56^dim^ and CD56^bright^ NK cell subsets to the initiation of inflammation and insulin resistance remain an open question (37). The role of numerous other NK cell subtypes also remains poorly defined. Data on circulating NK cell counts in T2D are inconsistent across studies (31); while the majority report no significant alterations, a few suggest increased frequencies of circulating NK cells accompanied by diminished cytotoxicity (69), whereas others observe a reduction in NK cell numbers (70). We observed no differences in the total number of NK cells or their subsets based on CD16 and CD56 expression in individuals with T2D (**Table 2**). Similarly, we found no significant differences in the proportions of NK cell subsets defined by CD8 and CD38 (**Table 2**), or NKG2A and CD161 (**Table 3**), when comparing T2D patients and healthy donors. However, most of the NK cell subsets that showed significant alterations in T1D patients also differed significantly from those in T2D patients (**Tables 2**, **3**), which is rather naturally explained considering the different type in inflammation that characterizes these two forms of diabetes. As a result of prolonged inflammation in both T1D and T2D, NK cells may undergo functional changes, leading to dysfunction and exhaustion (12, 24, 26). Early studies reported reduced cytotoxicity of peripheral blood NK cells in patients with T1D, which correlated with decreased numbers of NKG2D+ NK cells and increased numbers of KIR2DL3+ and NKG2A+ NK cells (71). In murine models of T1D, NK cells isolated from the spleen exhibited diminished cytotoxic activity, which declined further with disease progression (72). In our study, we observed a reduction in the frequency of granzyme B-containing CD3- cells in T1D (**Table 4**). Data on NK cell functional activity in T2D remain limited. Some reports describe impaired granzyme/perforin-mediated cytotoxicity of NK cells against tumor cells in obese patients. Others highlight phenotypic alterations in peripheral NK cells, characterized by elevated granzyme B and CD69 expression alongside decreased CD16, NKp46, and NKG2A+/CD94 expression. Additionally, some studies report no changes in baseline granzyme B/perforin production by NK cells in obesity (66). In our study, we found increased frequencies of perforin-containing CD56+ and granzyme B-containing CD16+ and CD56+ NK cells in T2D (**Table 4**). Interestingly, we also observed an increased proportion of CD8+EOMES+ NK cells in T2D (**Table 3**, **Figure 2I**), a subset with high cytotoxic potential. We attribute this elevated cytotoxic potential of NK cells in T2D to the relatively short disease duration in our cohort (**Table 1**).

NKT cells, which have diverged into a distinct lymphocyte lineage, play a pivotal role in immune defense (48). MAIT cells, identified as CD161++CD8+ T cells, constitute a small fraction of the total CD161++CD8+ T cell pool, with their numbers increasing with age and peaking around the third to fourth decade of life (73). Functionally, they resemble CD56^bright^ NK cells. In their resting state, both CD56^bright^ NK cells and CD161++CD8+ T cells (notably MAIT cells) exhibit limited cytolytic activity, lacking granzyme B expression and containing little perforin. However, following activation, they acquire potent cytotoxic capacity (74). Data on the role of MAIT cells in T1D remain scarce. Given that in transgenic NOD mice, MAIT cells have been shown to delay the onset of T1D, a protective function has been proposed (75). In our study, we observed a reduction in CD4+ MAIT cells in the T1D group (**Table 5**, **Figure 4F**), suggesting their migration to the pancreas.

A distinct role of NKT cells (or innate-like T-cells) and their subsets in maintaining metabolic homeostasis has been established (76). In patients with obesity complicated by T2D, their functional capacity appears impaired, with reduced secretion of IL-2 and IL-4, which are essential for the protective, anti-inflammatory function of these cells (particularly iNKT) in adipose tissue (77). The function of MAIT cells - whether pathogenic or protective - depends on the context of their activation (60). The role of these NKT cells, as well as other innate-like T-cells, may be dual in nature. On one hand, they help preserve tissue integrity by rapidly secreting cytokines in response to endogenous stress. Under physiological conditions, these cells support immune homeostasis and thermogenesis. On the other hand, chronic exposure to inflammatory cues - as observed in obesity and T2D - may drive them towards promoting inflammation. In our study, we observed a more than two-fold significant reduction in the frequency of MAIT cells among CD3+ T cells, and an almost significant trend towards reduction among CD8+ T cells (**Table 5**, **Figures 4B, D**) in T2D patients compared to both HD and individuals with T1D. Previous studies have reported a decrease in the DN MAIT cell subset in T2D (76, 77), though this was not observed in our patient cohort. It is hypothesized that MAIT cells are recruited from the circulation to the gut and/or adipose tissue in the context of T2D, leading to reduced frequencies in peripheral blood. Within adipose tissue, activated MAIT cells may produce IL-17 and contribute to inflammation.

iNKT cells are innate tissue-resident lymphoid T-cells that express an invariant TCR α-chain (Vα24-Ja18) and recognize CD1b, the MHC-I-class-like antigen-presenting molecule expressed on DCs and other APCs, which presents lipid and glycolipid antigens derived from microbial pathogens (49). Numerous studies using animal models have attributed a protective role to iNKT cells against T1D. An increase in their numbers significantly reduces the incidence of T1D in NOD mice (48, 76, 78). iNKT cells themselves represent a heterogeneous population. Certain subsets, such as iNKT17 cells, may in contrast exhibit pro-autoimmune properties. In humans, CD4+ and DN iNKT cells are functionally distinct, with the CD4+ subset serving as a primary source of Th2-type cytokines including IL-4. These subsets also display divergent patterns of chemokine receptor and integrin expression, influencing their migratory behavior (48). In T1D, a reduction in iNKT cell frequency has been observed among siblings of patients (48). However, reports regarding the frequency of circulating iNKT cells in patients with T1D remain inconsistent. Although findings from animal models regarding the role of iNKT cells in T2D remain contradictory, these cells are generally attributed a protective, anti-inflammatory function through the production of IL-4 and IL-10 (79). In our study, we observed no significant differences in the overall frequency of iNKT cells among total CD3+ T-cells across the groups; however, a reduction in CD4+ iNKT cells was noted in T2D (**Table 5**).

Finally, to identify possible associations between the studied immune cell populations and clinical parameters, we performed correlation analysis using Spearman’s rank correlation coefficients. Analysis of the statistically significant interrelationships among clinical parameters highlights the fundamental differences in the clinical marker profiles of T1D and T2D. The absence of a significant correlation between autoantibody titers and C-peptide levels in patients with T1D suggests that the degree of immune system activation does not directly reflect the residual insulin secretion capacity of β-cells. This supports the concept of heterogeneity in the rate of islet destruction and the age-related characteristics of apoptosis and regeneration (80, 81). In patients with T2D, the identified correlations fit logically within the pathophysiological framework of subclinical inflammation. The positive association between C-peptide levels and BMI reflects compensatory hyperinsulinaemia against a background of pronounced insulin resistance, primarily driven by visceral obesity (82). The association of elevated monocyte counts with hyperglycaemia and HbA1c, as well as their increase with disease duration, supports the role of activated myelopoiesis in sustaining chronic low-grade inflammation—a key component of T2D pathogenesis and a contributor to the heightened cardiovascular risk in this population (83). In contrast, healthy volunteers displayed a balanced immune profile, with harmonious distributions of leukocyte populations and an expected positive correlation between BMI and triglyceride levels, further emphasizing the specificity of the correlations observed in diabetes.

In the absence of data on the composition and distribution of NK cell populations within tissues, it is difficult to fully interpret the results of the correlation analysis between peripheral blood NK cell subsets and clinical parameters. NK cells and their subsets can migrate into inflammatory tissue sites, proliferate in response to local cytokines (such as IL−12 and IL−15), and then re-enter the peripheral circulation under the influence of systemic stimuli. A decrease in certain cell subsets in the periphery may reflect their migration to inflamed tissues—this could be the case for NK cells and their CD56^dim^CD16^bright^ subset in T1D (**Table 2**), or for MAIT cells and their subsets in both T1D and T2D (**Table 5**). The inverse correlation between CD4^+^ MAIT cells and C−peptide levels in T1D—given the known protective role of MAIT cells at early stages—may reflect the recruitment of CD4^+^ MAIT cells into the inflamed pancreatic islets (**Figure 6B**). The increase in peripheral CD8^+^ NK cells is more difficult to interpret; it is a possible consequence that a greater proportion of CD8^-^ NK cells with reduced cytotoxic potential have already migrated to the pancreas. We were unable to identify distinct patterns linking NK cell subsets to the titers of the five studied autoantibodies, each of which follows a unique trajectory of appearance and disappearance during T1D development and islet destruction. Nonetheless, a notable positive correlation between IA−2 autoantibodies and EOMES^+^ NK cells was observed. Since EOMES expression is critical for NK cell maturation and the acquisition of cytotoxic functions, the positive correlation with EOMES^+^ and inverse correlation with EOMES^-^ NK cells suggest a potentially diabetogenic role for EOMES^+^ NK cell subsets. Importantly, this positive correlation was observed in EOMES^+^ NK cell populations lacking inhibitory receptors CD161 and NKG2A. In T2D, we more frequently observed positive correlations between markers of inflammation and CD161^+^ NK cells, which may indicate a pro-inflammatory role for CD161^+^ NK cells in the context of T2D.

## Conclusion

The data obtained indicate an active role of NK cells and their numerous subsets in the pathogenesis of both T1D and T2D. In T1D, NK cells may contribute not only to direct tissue damage but also to impaired immune regulation. In adult-onset T1D at diagnosis, our findings suggest an immunoregulatory role for CD8+ NK cells. The observed increase in the CD8+CD161+ NK cell subset may contribute in protection of activated APCs from apoptosis via CD161 expression and consequently enhance the autoimmune response. Expression of NKG2A is known to suppress the cytolytic potential of NK cells against activated T cells including autoreactive clones, and we suggest that the expansion of the CD8+NKG2A+ subset in T1D may further intensify a diabetogenic immune response. At the same time, CD8 expression may protect NK cells themselves from activation-induced apoptosis. Elevated levels of CD8+CD38+ and CD8+EOMES+ NK cell subsets were also detected in T1D.

In contrast, in T2D characterized by chronic low-grade inflammation, NK cells exhibit a different profile. Given their key role in sensing metabolic stress and driving inflammation in adipose tissue, NK cells could actively contribute to metaflammation and its associated complications. However, in our study, we did not observe significant changes in the total NK cell population or in their CD56/CD16-defined subsets. Expression levels of CD8, CD38, and CD161 were also comparable to those in healthy controls. T2D was associated with more than a twofold reduction in the overall frequency of MAIT cells, with a trend toward decreased CD8+ MAIT cells. We hypothesize that in T2D, MAIT cells are recruited to the gut and/or adipose tissue, leading to their depletion in peripheral blood. In adipose tissue, activated MAIT cells may produce IL-17 and contribute to local inflammation.

In summary, both NK and NKT cells are undoubtedly involved in the pathogenesis of T1D and T2D, albeit via different mechanisms. In adult-onset T1D, the immunoregulatory influence of NK cells on other immune subsets appears to be central. In T2D, the role of conventional NK cells in metaflammation and its metabolic sequelae such as obesity, insulin resistance, and T2D is only beginning to be elucidated.

### Limitations of the study

It is well-known that glucose-lowering therapies can modulate immune readouts (e.g., receptor expression, trafficking, and downstream signaling). This applies to both T2D agents (metformin, sulfonylureas, SGLT2 inhibitors, GLP-1 receptor agonists, and, in some cases, basal insulin) and to insulin delivery strategies in T1D. To limit therapy-related confounding, we sampled relatively early in disease: T1D participants were within ≤1 year of diagnosis and uniformly on multiple daily injections with pens (basal–bolus; no pumps), and T2D participants had ≤1 year of disease (except for 2 patients with 2 and 4.5 years of disease duration). We further restricted inclusion to preserved renal function (estimated glomerular filtration rate (eGFR) >60 mL/min/1.73 m²) and observed acceptable glycemic control at sampling (median HbA1c 7.4% in T1D; 6.95% in T2D). Given that acute metabolic status is a dominant proximal determinant of immune tone, these features reduce, though cannot eliminate, the influence of medication class per se. We therefore interpret our findings as immune signatures arising under typical, guideline-concordant treatment rather than artificial, treatment-free states.

## Data Availability

The original contributions presented in the study are included in the article/**Supplementary Material**. Further inquiries can be directed to the corresponding author/s.
