# Supplementary material for: Changes in circulating NK and innate-like T cells in type 1 and type 2 diabetes
Source: Front Immunol. 2025 Dec 4;16:1667888. doi: 10.3389/fimmu.2025.1667888 (PMC12711767; doi:10.3389/fimmu.2025.1667888)
Supplement: Supplementary file 1 [file Table1.docx]

Supplementary Material

1. **Supplementary Materials And Methods**

**Polychromatic flow cytometry of NK cells**

Peripheral blood was collected from each patient into EDTA-containing tubes (ethylenediaminetetraacetic acid) as an anticoagulant. Samples were stored at room temperature prior to processing. Immunostaining with antibody panels was performed on the same day and within three hours of blood collection, prior to red blood cell lysis. For each patient, blood samples were stained using antibody mixtures assembled into three multicolour flow cytometry panels (Table 1). Optimal antibody titrations were determined prior to the study and are indicated alongside each antibody and clone in the respective panel descriptions (Table 1). Panel 1 was designed for the analysis of major lymphocyte subpopulations, including NK cells. Panel 2 focused on the characterization of NK and NKT cells. Panel 3 was used to assess the cytotoxic potential of NK cells.

**Table 1. List of antibodies used in flow cytometry panels**

|  | Antibody specificity | Clone | Dilution | Manufacture | Catalogue # |
| --- | --- | --- | --- | --- | --- |
| Panel 1 | CD4-BUV395 | SK3 | 1:70 | BD Biosciences (USA) | 563550 |
|  | CD38-BV421 | HB-7 | 1:70 | BioLegend (USA) | 356618 |
|  | CD45-V500 | HI30 | 1:100 | BD Biosciences (USA) | 560777 |
|  | CD8-BV605 | SK1 | 1:100 | BD Biosciences (USA) | 564116 |
|  | HLA-DR-BV650 | L243 | 1:50 | BioLegend (USA) | 307650 |
|  | CD16-PE-CF594 | 3G8 | 1:70 | BD Biosciences (USA) | 562293 |
|  | CD56-PE-Cy7 | MEM-188 | 1:70 | BioLegend (USA) | 304628 |
|  | CD3-APC | SP34-2 | 1:16 | BD Biosciences (USA) | 557597 |
|  | CD19-APC-H7 | SJ25C1 | 1:50 | BD Biosciences (USA) | 560177 |
| Panel 2 | CD4-BUV395 | SK3 | 1:70 | BD Biosciences (USA) | 563550 |
|  | TCR Vα7.2-BV421 | OF-5A12 | 1:50 | BD Biosciences (USA) | 749494 |
|  | CD45-V500 | HI30 | 1:100 | BD Biosciences (USA) | 560777 |
|  | CD8-BV605 | SK1 | 1:100 | BD Biosciences (USA) | 564116 |
|  | NKG2A – BV786 | 131411 | 1:50 | BD Biosciences (USA) | 747917 |
|  | TCRVδ2-FITC | B6 | 1:50 | BioLegend (USA) | 331406 |
|  | TCR Vα24-Jα18-PE | 6B11 | 1:50 | BioLegend (USA) | 342904 |
|  | CD16-PE-CF594 | 3G8 | 1:70 | BD Biosciences (USA) | 562293 |
|  | CD161-BB700 | HP-3G10 | 1:50 | BD Biosciences (USA) | 748239 |
|  | CD56-PE-Cy7 | MEM-188 | 1:70 | BioLegend (USA) | 304628 |
|  | EOMES-AF647 | X4-83 | 1:50 | BD Biosciences (USA) | 567168 |
|  | CD3-AF700 | SP34-2 | 1:70 | BD Biosciences (USA) | 557917 |
| Panel 3 | CD4-BUV395 | SK3 | 1:70 | BD Biosciences (USA) | 563550 |
|  | Granzyme B -BV421 | GB11 | 1:50 | BD Biosciences (USA) | 561142 |
|  | CD45-V500 | HI30 | 1:100 | BD Biosciences (USA) | 560777 |
|  | CD8-BV605 | SK1 | 1:100 | BD Biosciences (USA) | 564116 |
|  | CD69-BV786 | FN50 | 1:50 | BD Biosciences (USA) | 563834 |
|  | CD45RA-FITC | HI100 | 1:70 | BioLegend (USA) | 304106 |
|  | CD16-PE-CF594 | 3G8 | 1:70 | BD Biosciences (USA) | 562293 |
|  | Perforin-PerCP-Cy5.5 | δG9 | 1:50 | BD Biosciences (USA) | 563762 |
|  | CD56-PE-Cy7 | MEM-188 | 1:70 | BioLegend (USA) | 304628 |
|  | CD3-AF700 | SP34-2 | 1:70 | BD Biosciences (USA) | 557917 |

As the panels contained more than one antibody conjugated to a Brilliant Violet fluorochrome, all antibodies were diluted in Brilliant Stain Buffer (BD Biosciences, USA). A total of 50 μL of peripheral blood was incubated with the antibody mix, carefully resuspended, and incubated at 4°C for 30 minutes in the dark. After staining, erythrocytes were lysed using lysing buffer (FACS Lysing Solution, BD Biosciences, USA). The lysing buffer was removed, and the cells were additionally washed in PBS. At the final stage, 500 µl of PBS was added to the sample for panel 1, which was then measured on the flow cytometer. Intracellular staining with antibodies against EOMES in panel 2 and Perforin/Granzyme B in panel 3 was performed using different permeabilisation buffers. For panel 2, the Human FOXP3 buffer set (BD Biosciences, USA) was used according to the manufacturer’s protocol. A total of 0.5 ml of the working permeabilisation buffer was added to each tube and incubated in the dark for 30 minutes. Then, 1 ml of MACS buffer (PBS, 1% BSA, 2 mM EDTA) was added, mixed, and the cells were pelleted by centrifugation. The cell pellet was resuspended in 100 µl of MACS buffer, followed by the addition of anti-EOMES antibodies, and incubated for 30 minutes at room temperature in the dark. After staining, unbound antibodies were removed by washing in MACS buffer. Finally, 500 µl of MACS buffer was added and the sample was acquired. For panel 3, cell membrane permeabilisation was performed using Perm/Wash buffer (BD Biosciences, USA) according to the manufacturer’s instructions. A total of 1 ml of Perm/Wash buffer was added to the tube, mixed, and. The cell pellet was then resuspended in 100 µl of Perm/Wash buffer, anti-Perforin and anti-Granzyme B antibodies were added, and the mixture was incubated for 30 minutes at 4°C in the dark. After staining, unbound antibodies were removed by washing in Perm/Wash buffer. The final measurements were performed in 500 µl of MACS buffer.

Sample acquisition was performed using a BD LSRFortessa flow cytometer (BD Biosciences, USA), equipped with five lasers (355, 405, 488, 561, and 640 nm). Instrument performance was monitored and validated daily using BD Cytometer Setup and Tracking (CS&T) calibration beads (BD Biosciences, USA), which ensured that the target fluorescence intensity values for each channel remained within acceptable deviations. All samples were acquired at the rate not exceeding 2000 events per second, with complete acquisition of cells per tube. Stopping gate for 10,000 events was set in CD3-CD19- gate for panel 1, and in CD3- gate for panel 2 and 3. Spectral compensation was calculated using single-stained controls prepared either with BD anti-mouse Igκ compensation beads (BD Biosciences, USA), stained with the same fluorochrome-conjugated antibodies used in the panels, or with peripheral blood leukocytes. Compensation matrices were automatically generated with BD FACSDiva software (BD Biosciences, version 9.0.1) and minimally adjusted manually if required.

**Cytometry Data Gating Strategy**

Initially, all events were assessed on Time/FSC-H dot plot (Supplementary Figure 1A) to ensure signal consistency throughout data acquisition and to exclude potential turbulence in the cytometer fluidics caused by air intake from exhausted samples. Singlet events were subsequently gated on FSC-A/FSC-H dot plot (Supplementary Figure 1B) to eliminate cell doublets and aggregates from the analysis. These singlet events were then evaluated on CD45/SSC dot plot (Supplementary Figure 1C), where CD45-positive lymphocyte population was identified.

**Panel 1 – Analysis of Major Lymphocyte Subpopulations, Including NK Cells:**

Major lymphocyte subsets, including CD3+ T cells, CD19+ B cells, and CD3-CD19- NK cells, were identified from CD45+ lymphocytes using CD3-ABC/CD19-APC-H7 dot plot (Supplementary Figure 1D). CD3+ T cells were further examined to distinguish between CD4+ helper T cells and cytotoxic CD8+ T cells using CD4-BUV395/CD8-BV605 dot plot (Supplementary Figure 1E). CD3-CD19- NK cells were analysed for CD56 and CD16 expression using CD16-PE-Cy7/CD56-PE-CF594 dot plot, where six subtypes were identified: CD56^bright^CD16- (NK subtype 1), CD56^bright^CD16+/++ (NK subtype 2), CD56^dim^CD16^bright^ (NK subtype 3), CD56-CD16+/++ (NK subtype 4), CD56-CD16- (NK subtype 5), CD56^dim^CD16- (NK subtype 6) (Supplementary Figure 1F). In addition, CD3-CD19- NK cells were analysed for CD8 and CD38 expression using CD8-BV605/CD38-BV421 dot plot to identify four subpopulations: CD8-CD38+ NK cells, CD8+CD38+ NK cells, CD8+CD38- NK cells, CD8-CD38- NK cells (Supplementary Figure 1G). Furthermore, within CD3-CD19- NK cells, selected subpopulations – namely all CD56^bright^CD16- NK cells (subtype 1) and CD56-CD16++ NK cells – were additionally analysed for CD8 and CD38 expression (Supplementary Figure 1H,I).

| **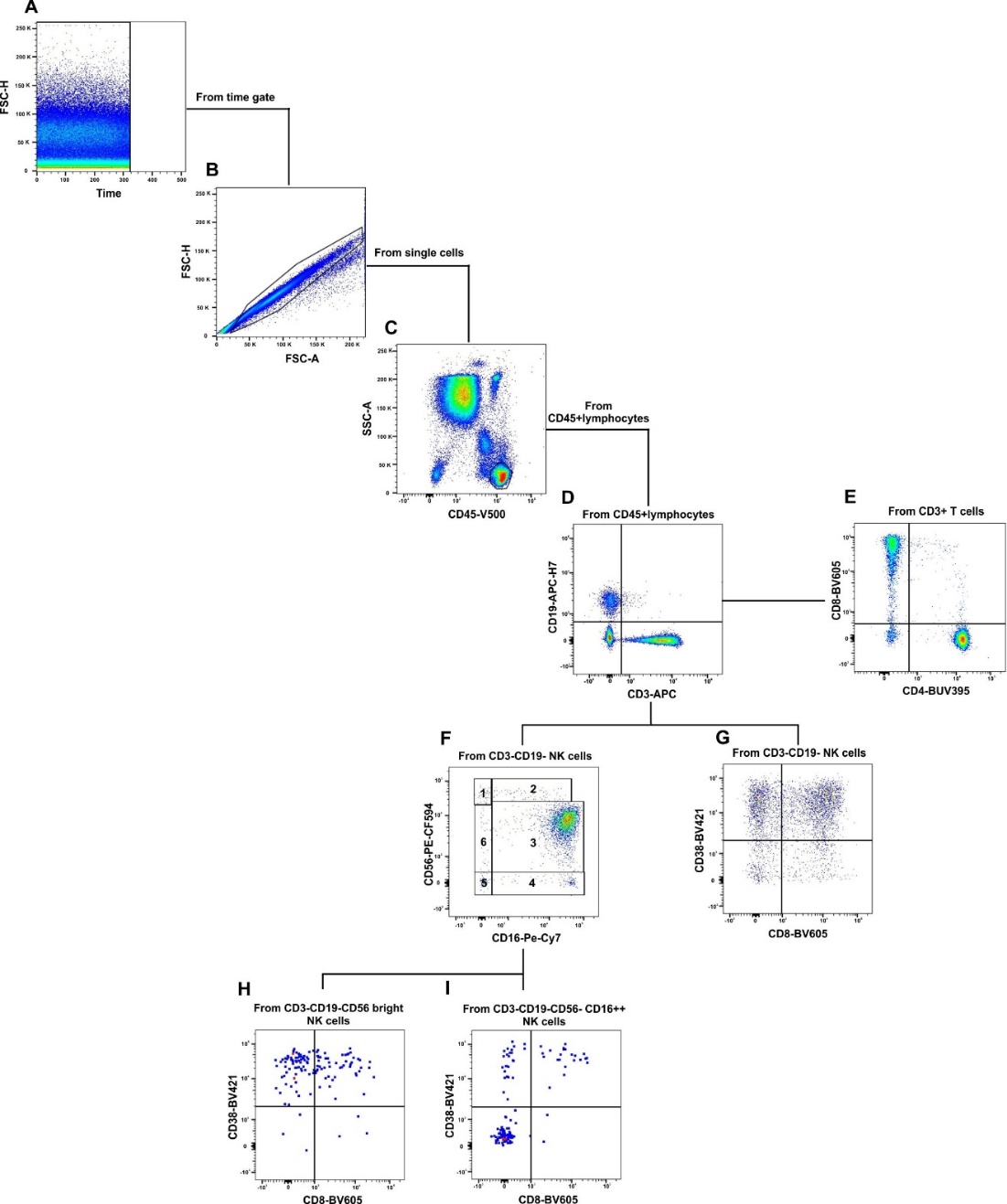** |
| --- |
| Supplementary Figure 1. Gating strategy for NK cells and major lymphocyte subpopulations based on antibody panel 1. (A-C) Identification of CD45-positive lymphocytes (А) following gating by time (B) and exclusion of cell aggregates (C). (D) Delineation of CD3+ T cells, CD19+ B cells and CD3-CD19- NK cells from CD45+ lymphocytes using CD3-ABC/CD19-APC-H7 dot plot. (E) Determination of circulating CD4+ Th cells and cytotoxic CD8+ T cells among CD3+ T cells using CD4-BUV395/CD8-BV605 dot plot. (F) Analysis of NK cell subpopulations identified within CD3-CD19- NK cell gate by CD56 and CD16 expression, distinguishing six subtypes: CD56^bright^CD16- NK subtype 1, CD56^bright^CD16+/++ NK subtype 2, CD56^dim^CD16^bright^ NK subtype 3, CD56-CD16+/++ NK subtype 4, CD56-CD16- NK subtype 5, CD56^dim^CD16- NK subtype 6. (G) Analysis of NK cell subpopulations (from CD3-CD19- gate) by CD8 and CD38 expression, identifying four subsets: CD8-CD38+ NK cells, CD8+CD38+ NK cells, CD8+CD38- NK cells and CD8-CD38- NK cells. (H-I) Assessment of CD8 and CD38 expression in CD56^bright^СD16- subtype 1 and CD56-CD16++ subtype 4 NK cells. |

**Panel 2 for NK and NKT cell analysis:**

As with other panels, initial gating was performed by time, followed by identification of singlets and CD45+ lymphocytes. NK cells were initially identified as CD3-negative events on CD3-AF700/SSC dot plot (Supplementary Figure 2A), from which the total NK cell population was more precisely gated on a CD16-PE-Cy7/CD56-PE-CF594 dot plot, excluding CD16-CD56- double-negative (DN) events for the further analysis (Supplementary Figure 2B). NK cells were then analysed for co-expression of several surface markers: CD8, CD161, NKG2A and EOMES using the following dot plots: CD161-BB700/CD8-BV605 (Supplementary Figure 2C), CD8-BV605/NKG2A-BV786 (Supplementary Figure 2D), CD161-BB700/NKG2A-BV786 (Supplementary Figure 2E), CD8-BV605/EOMES-AF647 (Supplementary Figure 2F), CD161-BB700/EOMES-AF647 (Supplementary Figure 2G), and EOMES-AF647/NKG2A-BV786 (Supplementary Figure 2H).

This antibody panel also allowed for the identification of specific NKT cell subpopulations: MAIT and iNKT. The overall MAIT and iNKT populations among total CD3+ T cells were gated using TCR Vα7.2-BV421/TCR Vα24-Jα18-PE dot plot (Supplementary Figure 2I). CD161-BB700/TCR Vα7.2-BV421 dot plot was used to assess the frequency of MAIT cells among cytotoxic CD8+ T cells (Supplementary Figure 2J), CD4+ Th cells (Supplementary Figure 2K), and CD4-CD8- DN T cells (Supplementary Figure 2L), by displaying each respective T cell subset. Similarly, CD161-BB700/TCR Vα24-Jα18-PE dot plot was used to determine the frequency of iNKT cells among cytotoxic CD8+ T cells (Supplementary Figure 2M), CD4+ Th cells (Supplementary Figure 2N), and CD4-CD8- DN T cells (Supplementary Figure 2O), again by displaying each respective T cell subset.

| 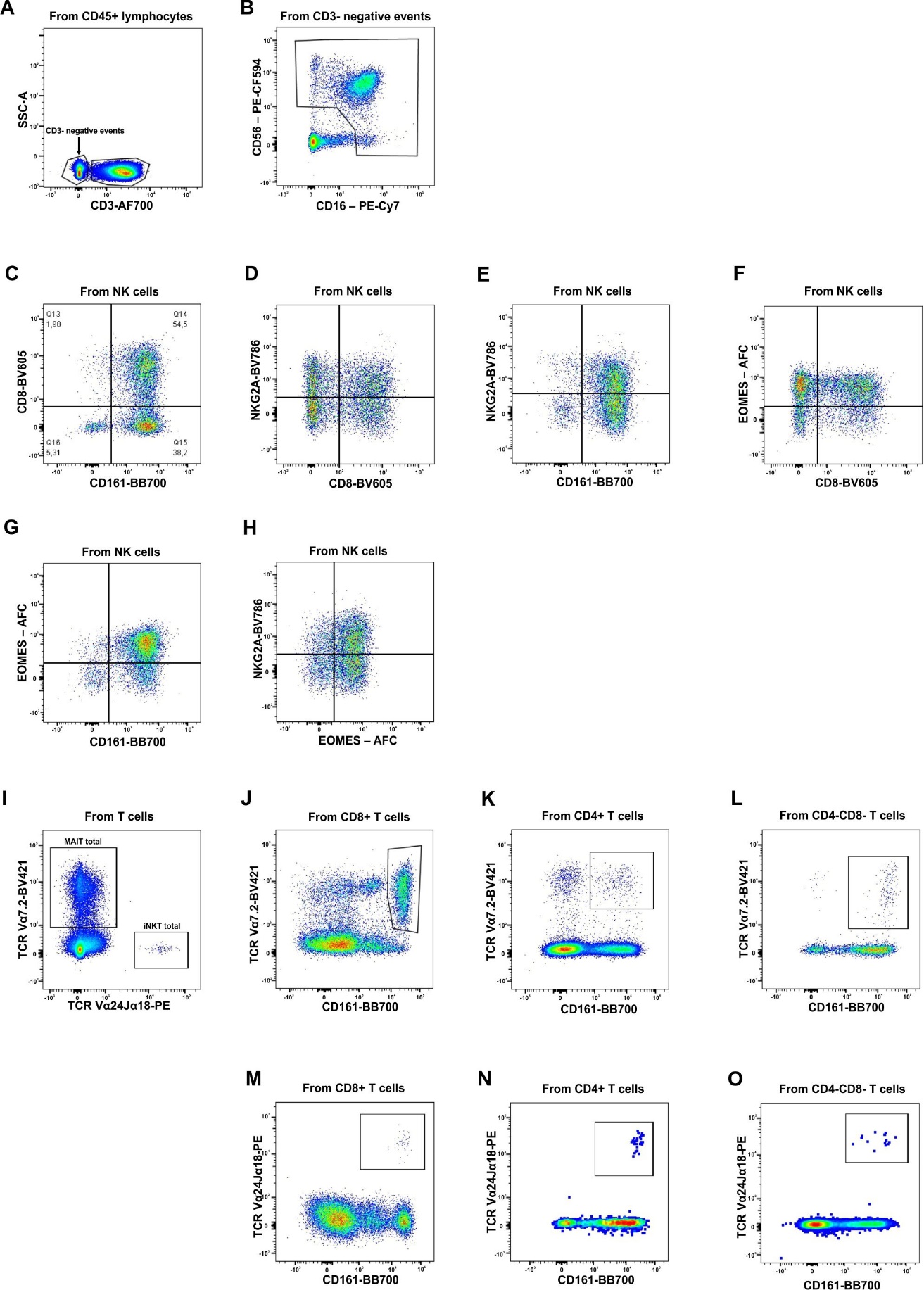 |
| --- |
| Supplementary Figure 2. Gating strategy for NK cell subpopulations based on CD8, CD161, NKG2A and EOMES expression, and for invariant NKT cells, using antibody panel 2. (A) Identification of CD3-negative events on CD3-AF700/SSC dot plot gated from total CD45+ lymphocytes. (B) Gating of NK cells on CD16-PE-Cy7/CD56-PE-CF594 dot plot; (C-H) Dot plots used for analysing NK cells based on co-expression of CD8, CD161, NKG2A and EOMES. (I) TCR Vα7.2-BV421/TCR Vα24-Jα18-PE dot plot displaying total CD3+ T cells, used to identify overall MAIT and iNKT populations among CD3+ T cells. (J-L) CD161-BB700/TCR Vα7.2-BV421 dot plots displaying cytotoxic CD8+ T cells (J), CD4+ Th cells (K), and CD4-CD8- double-negative (DN) T cells (L), for identification of MAIT cells within each subset. (M-O) CD161-BB700/TCR Vα24-Jα18-PE dot plots displaying cytotoxic CD8+ T cells (M), CD4+ Th cells (N), and CD4-CD8- DN T cells (O), for identification of iNKT cells within each subset. |

**Panel 3 for assessing NK cell cytotoxic potential:**

As with the other panels, initial gating included time gating, singlet discrimination, and identification of CD45+ lymphocytes. NK cells were first defined as CD3-negative events on the CD3-AF700/SSC dot plot (Supplementary Figure 3A), from which the total NK cell population was more precisely gated on CD16-PE-Cy7/CD56-PE-CF594 plot, excluding CD16-CD56- double-negative events from analysis (Supplementary Figure 3B). To calculate perforin potential, all CD16+/CD56+ NK cells identified in the previous gating step were analysed on CD16-PE-Cy7/Perforin-PerCP-Cy5.5 (Supplementary Figure 3C) and CD56-PE-CF594/Perforin-PerCP-Cy5.5 (Supplementary Figure 3D) dot plots. Perforin potential for CD16+ NK cells was calculated using the formula: (CD16+Perforin+) / ((CD16+Perforin+) + (CD16+Perforin-)) × 100%. The analogous formula was used to calculate perforin potential for CD56+ NK cells. For granzyme potential, all CD16+/CD56+ NK cells were evaluated on the dot plots CD16-PE-Cy7/Granzyme B–BV421 (Supplementary Figure 3E) and CD56-PE-CF594/Granzyme B–BV421 (Supplementary Figure 3F). Granzyme potential was calculated in the same manner as perforin potential. To assess perforin and granzyme potential within CD8+ NK cells, the entire NK cell population was analysed on the dot plots CD8-BV605/Perforin-PerCP-Cy5.5 (Supplementary Figure 3G) and CD8-BV605/Granzyme B–BV421 (Supplementary Figure 3H), respectively. In addition, the proportion of perforin- and granzyme B-positive events among CD3- cells was evaluated from the total CD45+ lymphocytes on the dot plots CD3-AF700/Perforin-PerCP-Cy5.5 (Supplementary Figure 3I) and CD3-AF700/Granzyme B–BV421 (Supplementary Figure 3J). The activation status of NK cells was assessed based on CD69 and CD25 expression using the dot plots CD3-AF700/CD69-BV786 (Supplementary Figure 3K) and CD3-AF700/CD25-PE (Supplementary Figure 3L), by determining the proportion of CD3-CD69+ and CD3-CD25+ cells, respectively. Additionally, the frequency of CD8+CD69+ NK cells was assessed using the CD8-BV605/CD69-BV786 dot plot applied to the total NK cell population (Supplementary Figure 3M).

| 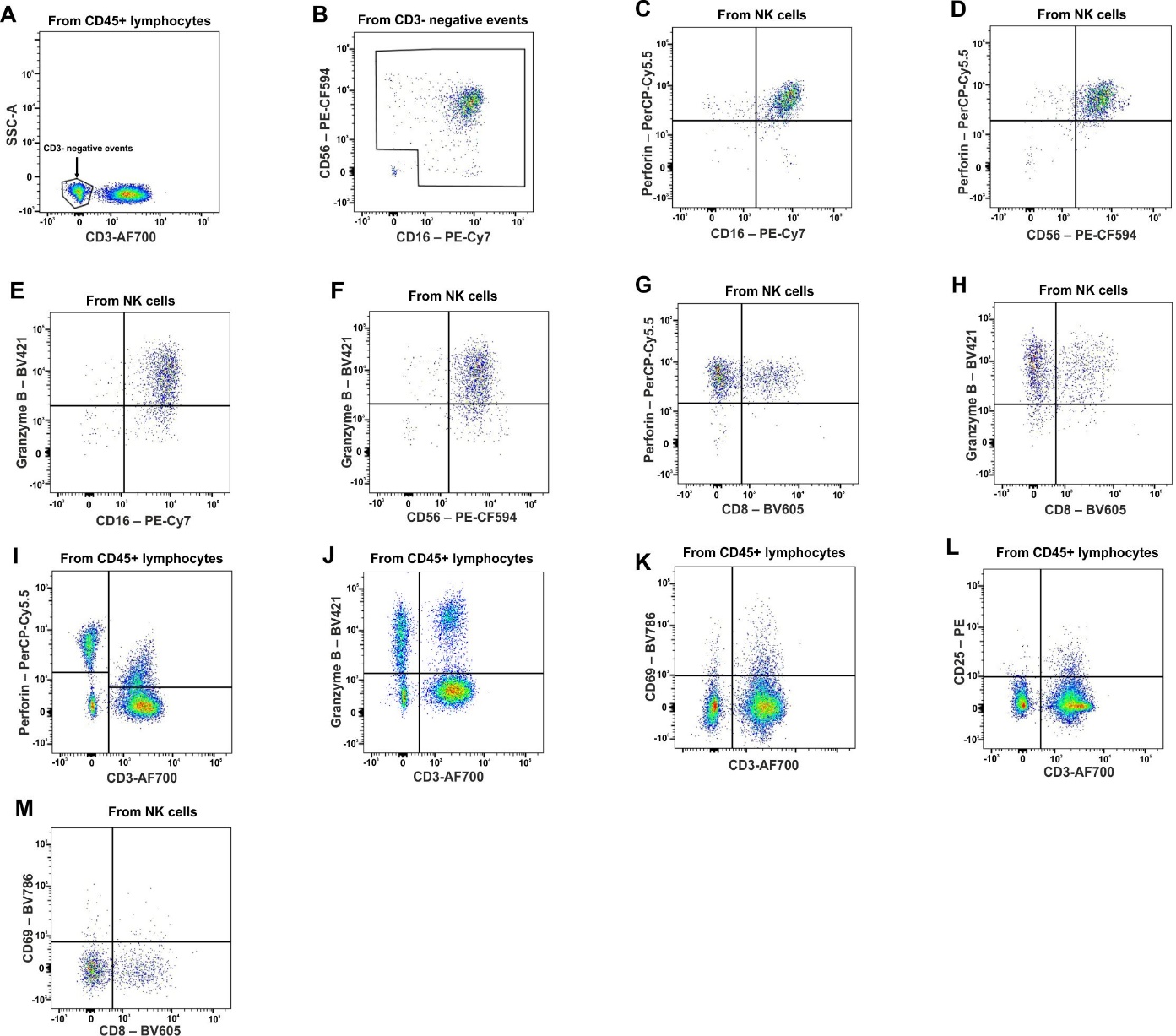 |
| --- |
| Supplementary Figure 3. Identification of NK cells potentially capable of secreting granules with perforin and granzyme B. (A) Identification of CD3-negative events on the dot plot CD3-AF700/SSC from all CD45+ lymphocytes. (B) Identification of NK cells on the dot plot CD16-PE-Cy7/CD56-PE-CF594. (C-D) Dot plots CD16-PE-Cy7/Perforin-PerCP-Cy5.5 (C) and CD56-PE-CF594/Perforin-PerCP-Cy5.5 (D), showing all previously gated NK cells for the calculation of perforin potential in CD16+ and CD56+ NK cells. (E-F) Dot plots CD16-PE-Cy7/Granzyme B–BV421 (E) and CD56-PE-CF594/Granzyme B–BV421 (F), showing all NK cells for the calculation of granzyme potential in CD16+ and CD56+ NK cells. (G-H) Dot plots CD8-BV605/Perforin-PerCP-Cy5.5 (G) and CD8-BV605/Granzyme B–BV421 (H), showing all NK cells for the calculation of perforin and granzyme potential in CD8+ NK cells. (I-J) Dot plots CD3-AF700/Perforin-PerCP-Cy5.5 (I) and CD3-AF700/Granzyme B–BV421 (J), showing all CD45+ lymphocytes. (K-L) Dot plots CD3-AF700/CD69-BV786 (K) and CD3-AF700/CD25-PE (L) for the assessment of activation status of CD3- NK cells. D3 - dot plot CD8-BV605/CD69-BV786 showing all NK cells for evaluating the proportion of CD8+CD69+ NK cells. |
| **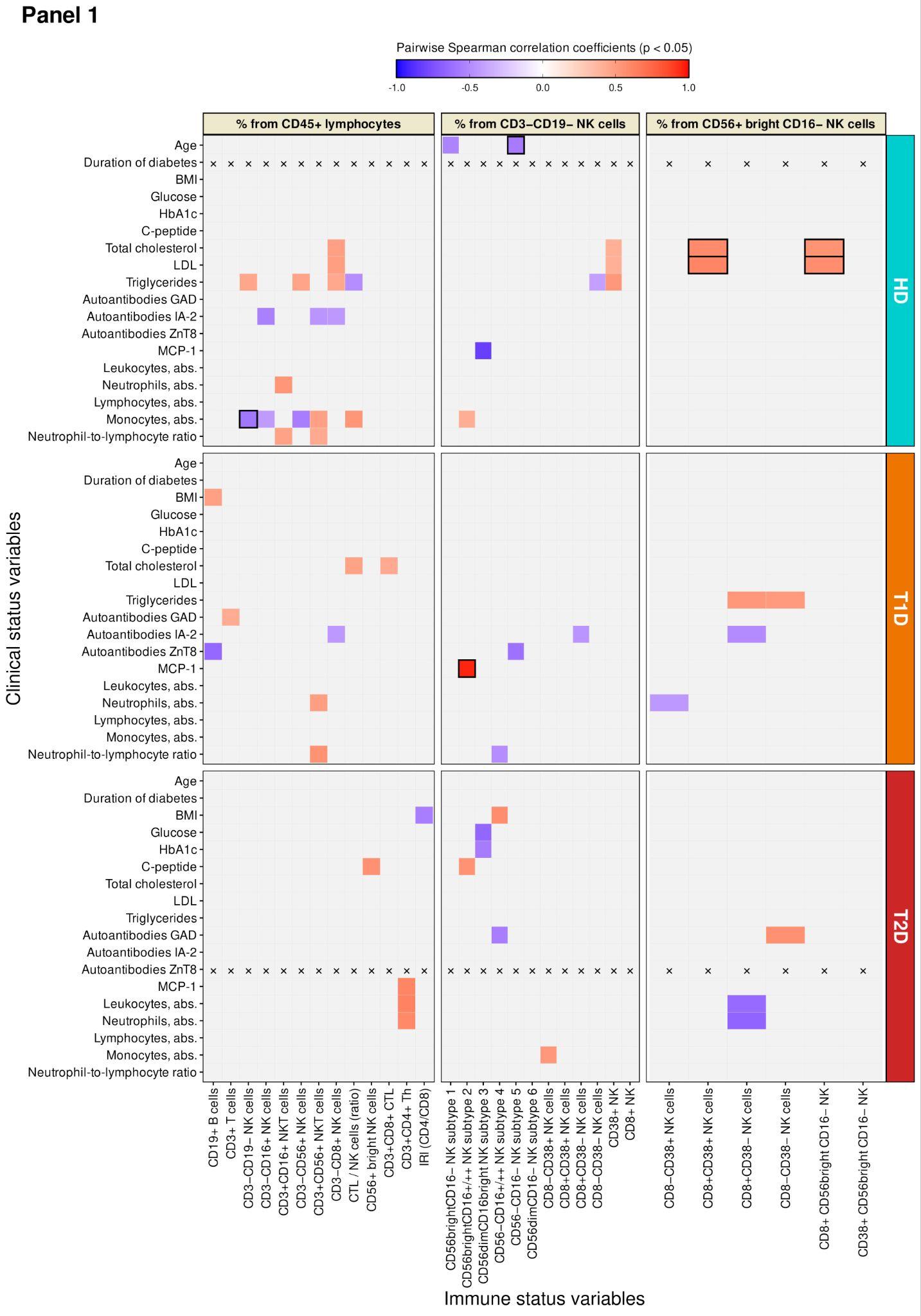** |
| Supplementary Figure 4. Heatmap of correlations between clinical parameters and immune cell subsets identified using Antibody Panel 1. Heatmap cells with statistically significant correlations at p < 0.05 are colored and the cells with significance level of p < 0.005 are additionally outlined with black borders. The cells with insignificant correlations (p > 0.05) are marked with gray color. The cells with missing or not applicable data are labeled with symbol "x". |

| 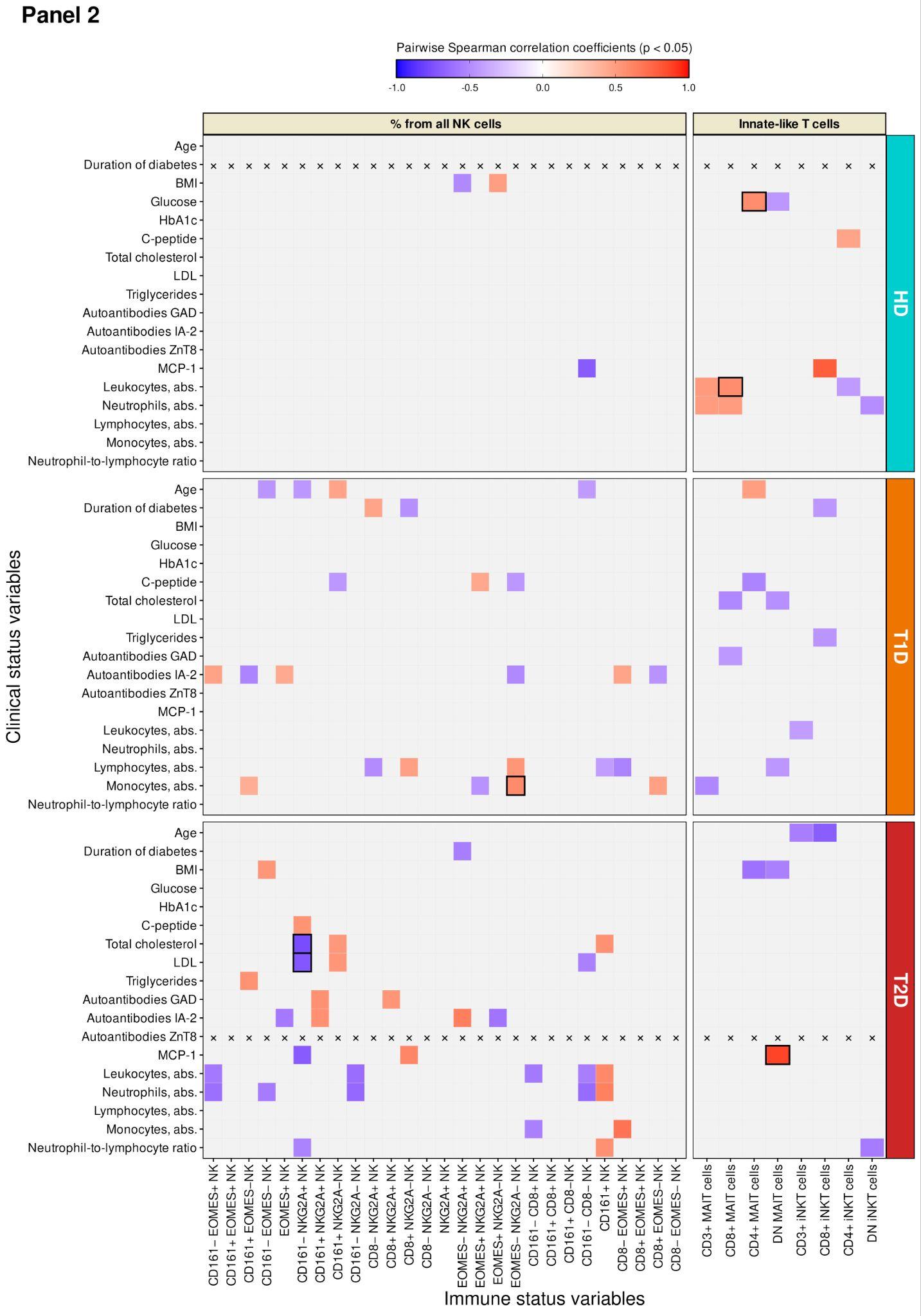 |
| --- |
| Supplementary Figure 5. Heatmap of correlations between clinical parameters and immune cell subsets identified using Antibody Panel 2. Heatmap cells with statistically significant correlations at p < 0.05 are colored and the cells with significance level of p < 0.005 are additionally outlined with black borders. The cells with insignificant correlations (p > 0.05) are marked with gray color. The cells with missing or not applicable data are labeled with symbol "x". |
| **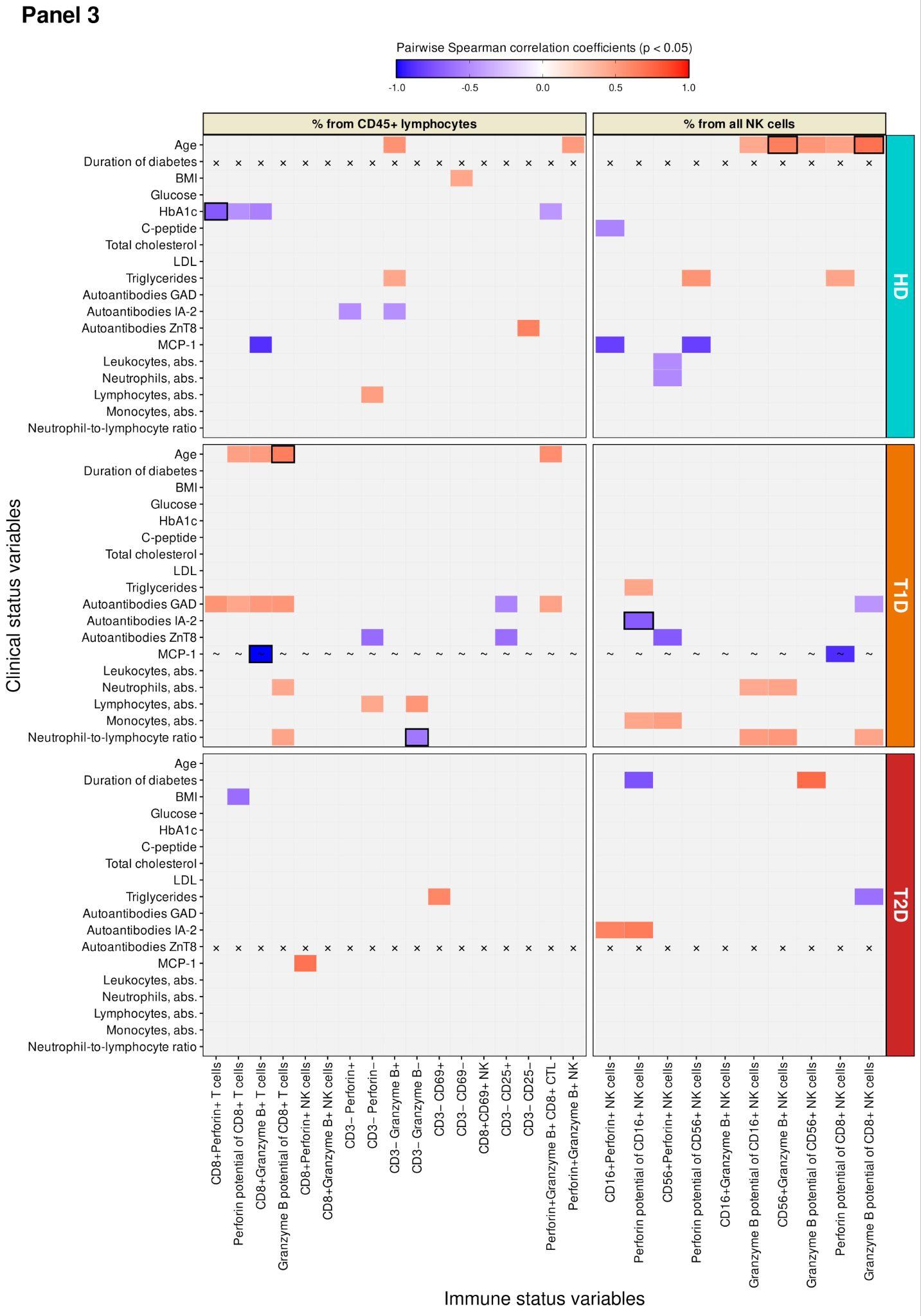** |
| Supplementary Figure 6. Heatmap of correlations between clinical parameters and immune cell subsets identified using Antibody Panel 3. Heatmap cells with statistically significant correlations at p < 0.05 are colored and the cells with significance level of p < 0.005 are additionally outlined with black borders. The cells with insignificant correlations (p > 0.05) are marked with gray color. The cells with missing or not applicable data are labeled with symbol "x", the cells corresponding to a small number of paired measurements (n ≤ 5) are marked with symbol **"~".** |

| 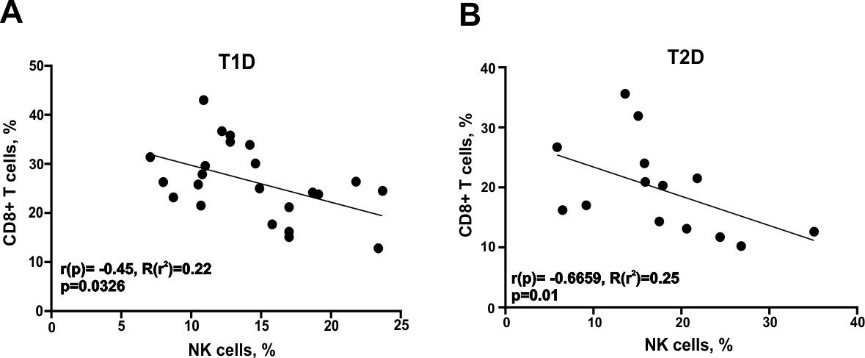 |
| --- |
| Supplementary Figure 7. Some Immune-Immune correlations. (A) Correlation between the frequency of NK cells and cytotoxic CD8+ T cells in T1D. (B) Correlation between the frequency of NK cells and cytotoxic CD8+ T cells in T2D. |
